# Revolving SEM images visualising 3D taxonomic characters: application to six species of the millipede genus *Ommatoiulus* Latzel, 1884, with description of seven new species and an interactive key to the Tunisian members of the genus (Diplopoda, Julida, Julidae)

**DOI:** 10.3897/zookeys.328.5763

**Published:** 2013-09-03

**Authors:** Nesrine Akkari, David Koon-Bong Cheung, Henrik Enghoff, Pavel Stoev

**Affiliations:** 1 Natural History Museum of Denmark, University of Copenhagen, Universitetsparken 15, DK-2100 København Ø, Denmark; 2 National Museum of Natural History, Bulgarian Academy of Sciences; 3 Pensoft Publishers, 12, Prof. Georgi Zlatarski St., 1700 Sofia, Bulgaria

**Keywords:** Taxonomy, millipedes, Diplopoda, *Ommatoiulus*, new species, North Africa, interactive SEM images, interactive key

## Abstract

A novel illustration technique based on scanning electron microscopy is used for the first time to enhance taxonomic descriptions. The male genitalia (gonopods) of six species of millipedes are used for construction of interactive imaging models. Each model is a compilation of a number of SEM images taken consecutively while rotating the SEM stage 360°, which allows the structure in question to be seen from all angles of view in one plane. Seven new species of the genus *Ommatoiulus* collected in Tunisia are described: *Ommatoiulus chambiensis*, *Ommatoiulus crassinigripes*, *Ommatoiulus kefi*, *Ommatoiulus khroumiriensis*, *Ommatoiulus xerophilus*, *Ommatoiulus xenos*, and *Ommatoiulus zaghouani* **spp. n.** Size differences between syntopic adult males of *Ommatoiulus chambiensis* and *Ommatoiulus xerophilus* **spp. n.** from Châambi Mountain are illustrated using scatter diagrams. A similar diagram is used to illustrate size differences in *Ommatoiulus crassinigripes*, *Ommatoiulus khroumiriensis* **spp. n.** and *Ommatoiulus punicus* (Brölemann, 1894). In addition to morphological differences, the latter three species display allopatric distribution and different habitat preferences. A dichotomous interactive key with a high visual impact and an intuitive user interface is presented to serve identification of the 12 *Ommatoiulus* species so far known from Tunisia. Updates on the North African *Ommatoiulus* fauna in general are presented.

## Introduction

Description of new species is just one among many tasks of taxonomists (Enghoff and Seberg 2006), but this task is becoming increasingly urgent due to the continuing global decline of biodiversity. However, descriptive taxonomy has a big problem with keeping up to speed. Thus, it has recently been estimated that it takes on average 21 years for a species from being discovered and collected to be formally named and described (Fontaine et al. 2012). The ‘shelf life’ was, for example, ca. 150 years for *Ommatoiulus schubarti* Akkari & Enghoff, 2012, a species collected for the first and hitherto only time in 1863 (Akkari and Enghoff 2012).

Enhancing and modernizing taxonomy constitutes one of the main challenges of this century, and several pilot projects and initiatives have been taken in this respect (see La Salle et al. 2009, Deans et al. 2012, Erwin et al. 2012). Nevertheless, there is still scope for improvement of efficiency. Costello et al. (2013) listed 14 “Actions to increase the species description rates and taxonomic efficiency”, including “Use of digital imaging and molecular technologies to accelerate the description of species” and “Increased availability and access to museum and herbarium specimens, particularly type specimens through exchanges, loans, and on-line imaging...”.

It is natural that the word “imaging” appears in two of Costello et al.’s 14 action points, because nowhere is the saying “a picture is worth a thousand words” more true than in taxonomic descriptions. Early taxonomic works often included excellent drawings. In the course of time, drawings have been supplemented with photographs, microphotographs, SEM micrographs, multi-focus images, and (most recently) images produced by confocal laser scanning microscopy (cLSM), optical projection tomography (OPT), magnetic resonance imaging (MRI) and micro-Computed Tomography scan (e.g., Heim and Nickel 2010, Błażejowski et al. 2011, Görög et al. 2012, Faulwetter et al. 2013). The latter authors in particular have made a significant contribution in this field, suggesting that virtual specimens prepared by means of micro-CT scan may replace type specimens for some purposes.

To demonstrate a new technique for visualization of taxonomic characters described in detail in (Cheung et al. 2013) we use six species of the millipede genus *Ommatoiulus* Latzel, 1884 from Tunisia. We describe 7 new *Ommatoiulus* species and offer an interactive and highly visual key to all 12 *Ommatoiulus* species from Tunisia for users with a suitable browser plug-in or Flash viewer.

This work is part of an ongoing project of revising the tribe Schizophyllini (Akkari and Enghoff 2011, 2012, Akkari 2013).

## Taxonomic characters in millipedes and associated problems

The male copulatory organs, or gonopods, are of prime importance for characterising millipede species and higher taxa. There are exceptions, where different species have identical or almost identical gonopods, e.g., several genera of Juliformia, such as *Nepalmatoiulus* Mauriès, 1983 (Enghoff 1987), *Dolichoiulus* Verhoeff, 1900 (Enghoff 1992), *Pachyiulus* Berlese, 1883 (Frederiksen et al. 2012), *Anadenobolus* Silvestri, 1897 (Bond and Sierwald 2002) and *Thyropygus* Pocock, 1894 (Pimvichai et al. 2011a, b), and of Nematophora, e.g., *Sinocallipus* Zhang, 1993 (Stoev and Enghoff 2011). By and large, however, the gonopods carry enough information to recognize species, and for more than 150 years authors have focused on describing and illustrating these structures in the most reliable way (Highton 2009).

As useful as gonopod illustrations are for taxonomic descriptions, they can sometimes be grossly misleading and result in misidentification and production of synonymic names, the Achilles’ heel of descriptive taxonomy. There are several examples of this in the literature about millipedes, but most striking is perhaps the case presented by Hauser (2000) who demonstrated that amongst 11 subspecies and 100 varieties described for *Craspedosoma alemannicum* Verhoeff, 1910 (see Schubart 1963a), based on differences in the length of podosternite (posterior gonopods) processes, only 9% are valid while the rest can be discarded. The reason for this is that the ‘heterodactyly’ on which Verhoeff (1915, 1916, 1917, 1939) based his infraspecific *Craspedosoma* taxonomy and which was subsequently adopted by most authors studying the taxonomy and ecology of the genus, was simply due to observation error. When the podosternite of *Craspedosoma* is viewed from varying angles, the relative lengths of its processes change (see Hauser 2000, figs 1, 2, 3).

Useful taxonomic characters in *Ommatoiulus* are almost exclusively derived from the gonopods. Differences between species are often subtle, and the pronouncedly “3D” nature of the gonopods makes recognition of the differences difficult. In many older papers dealing with *Ommatoiulus* taxonomy, authors have dissected the different parts of the gonopods and have illustrated them separately which has led not only to “angle-of-view” problems, but also to difficulties of relating the various gonopod components spatially to each other. By applying the novel imaging technique we have overcome these problems.

### The study group: *Ommatoiulus* millipedes from Tunisia

*Ommatoiulus* is the dominant genus of julid millipedes in North Africa and the Iberian Peninsula. A total of 70 species have been described so far, and many more remain to be recognised and named. For example, Akkari and Enghoff (2012) recorded 19 *Ommatoiulus* species in the southernmost Spanish region, Andalusia, 10 of which they described as new. These authors further provided a historical overview tracing the general inconclusive taxonomic situation and gave an updated definition of the genus based on morphological characters and a key to the 19 Andalusian species which they estimated to constitute at most 1/6 of the total species richness for the genus. In spite of the wide distribution of a few species, e.g. *Ommatoiulus sabulosus* (Linnaeus, 1758) reaching 64°N in Fennoscandia, and *Ommatoiulus moreleti* (Lucas, 1860) with a near-cosmopolitan, synanthropic distribution, most *Ommatoiulus* species are confined to the Mediterranean region of North Africa and Iberia, and tend even to display small-scale endemism. For instance,of the 19 species recorded from Andalusia, only five were found in other areas (Akkari and Enghoff 2012).

North African species of the genus were examined in considerable detail by several authors, especially Brolemann (1921, 1924, 1925a, b) and Schubart (1952, 1960, 1963b). Recent studies have mostly targeted the Tunisian fauna, describing new species (Akkari and Voigtländer 2007, Akkari and Enghoff 2011) and in some cases detailing some aspects of developmental modalities (Akkari and Enghoff 2011). Akkari et al. (2009) presented detailed species accounts and new records from Tunisia in addition to a complete bibliographical review of the order Julida in North Africa, listing 24 *Ommatoiulus* species for the region.

Despite these contributions, the North African *Ommatoiulus* fauna is far from being thoroughly assessed, nor is its taxonomy close to being fully revised. Without doubt, numerous new species still await discovery, and several taxonomic questions still remain unsolved, such as the correct placement of the highly deviating species *Ommatoiulus lapidarius* (Lucas, 1846), type species of the subgenus *Apareiulus* Brölemann, 1897 (e.g., Attems 1952, Schubart 1963b). The same applies to the *Ommatoiulus punicus* species group established to facilitate understanding species affinities (Akkari and Voigtländer 2007) but which, in the light of recent revisionary work on the genus (Akkari and Enghoff 2012), might not reflect true relationships. To solve these questions an exhaustive revision of the genus in this area is needed.

## Material and methods

Most specimens were hand collected during spring 2008 by N.A. and P.S. Supplementary material was obtained from museum collections. All studied specimens are preserved in 70% alcohol. Measurements were made using a Leica Wild M10 microscope equipped with an ocular micrometre. Photographs were taken using Visionary Digital’s BK Plus Lab with a Canon EOS 7D. For scanning electron microscopy, parts of the specimens were cleaned with ultrasound, transferred to 96% ethanol then to acetone, air-dried, mounted on adhesive electrical tape attached to aluminium stubs, coated with platinum/palladium and studied in a JEOL JSM-6335F scanning electron microscope. Photographs were processed with a Leica Application Suite program and final stacking made with Zerene Stacker 1.04. The rotatable images were constructed from 18 SEM images taken at 20 degrees intervals starting from the mesal view and continuing until all 360 degrees were captured by rotating the SEM stage. The images were processed using Adobe Lightroom 4.3 by adjusting the black, highlight and white levels to achieve a uniform background and contrast. Each image was then cropped to ensure a smooth transition between each frame during rotation. The images were imported into Adobe Flash CS5, where each image was made into a single frame and the series combined to form a rotating animation. The animation controls (moving from one frame to the next) were mapped to the mouse cursor using Actionscript 3.0. The html version available online was compiled using Magic 360. The interactive key was developed in Adobe Flash CS5.5 using Actionscript 2.0 to handle screen transitions and image swapping. Plates were assembled using Adobe Indesign CS 5.5. Respective image libraries of the interactive rSEM have Been Deposited In Morphbank. More details on the method of creation of the interactive models can be found in Cheung et al. (2013). The number of body rings is given as recommended by Enghoff et al. (1993): Number of podous rings (PR) + number of apodous rings (AR) + telson (T). The developmental stadium of a number of individuals was taken as being represented by the number of vertical rows of ocelli (RO). The real stadium number in julid millipedes has been shown to equal the number of RO+1 (Enghoff et al. 1993), e.g., a specimen with 9RO belongs to developmental stadium 10.

### Abbreviations

ARapodous rings

Hvertical midbody diameter (height)

Lbody length

MNHNMuséum National d’histoire Naturelle, Paris

MSNBMuseo Civico di Storia Naturale ‘Enrico Caffi’, Bergamo, Italy

NMNHSNational Museum of Natural History, Sofia

PRpodous rings

ROvertical rows of ocelli

Ttelson

ZMUCNatural History Museum of Denmark (Zoological Museum), University of Copenhagen

## Results

### Order Julida Brandt, 1833
Family Julidae Leach, 1814
Tribe Schizophyllini Verhoeff, 1909

#### Genus Ommatoiulus

Latzel, 1884

http://species-id.net/wiki/Ommatoiulus

##### Remarks.

Ageneral characterisation of *Ommatoiulus* was given by Akkari and Enghoff (2012). Delimitation of *Ommatoiulus* vis-à-vis related nominal genera such as *Tachypodoiulus* Verhoeff, 1893 and *Rossiulus* Attems, 1926 is not very clear at present and will probably remain so until the ongoing comprehensive revision of Schizophyllini has been completed (see Akkari and Enghoff 2012).

Of the ca. 70 species of *Ommatoiulus* currently recognized, the following occur in Tunisia:

*Ommatoiulus chambiensis* Akkari & Enghoff, sp. n.

*Ommatoiulus crassinigripes* Akkari & Enghoff, sp. n.

*Ommatoiulus fuscounilineatus* (Lucas, 1846)

*Ommatoiulus kefi* Akkari & Enghoff, sp. n.

*Ommatoiulus khroumiriensis* Akkari & Enghoff, sp. n.

*Ommatoiulus malleatus* Akkari & Voigtländer, 2007

*Ommatoiulus punicus* (Brölemann, 1894)

*Ommatoiulus sempervirilis* Akkari & Enghoff, 2011

*Ommatoiulus seurati* (Brolemann, 1925)

*Ommatoiulus xenos* Akkari & Enghoff, sp. n.

*Ommatoiulus xerophilus* Akkari & Enghoff, sp. n.

*Ommatoiulus zaghouani* Akkari & Enghoff, sp. n.

#### Ommatoiulus chambiensis

Akkari & Enghoff sp. n.

http://zoobank.org/C6950942-CCF3-43CA-96FF-75F9166168ED

http://species-id.net/wiki/Ommatoiulus_chambiensis

Figs 1 –6

##### Material.

**Holotype**: ♂, W Tunisia, Kasserine Governorate, Châambi National Park, surroundings of the park´s guest house, 35°10.139'N, 8°40.486'E, alt. 950–1000m, scarce trees, *Pinus halepensis*, under stones, 7.3.2008, P. Stoev & N. Akkari leg. (ZMUC). **Paratypes**: 17 ♂♂, 31 ♀♀, 1 immature, same data as holotype (ZMUC); 2 ♂♂, 2 ♀♀, same data as holotype (NMNHS); 1 ♂, 2 ♀♀, W Tunisia, Kasserine Governorate, Châambi National Park, 35°11. 901'N, 8°39.505'E, alt. 1291m, *Quercus ilex*, *Pinus halepensis*, slope, under stones and in leaf litter, 9.3.2008, P. Stoev & N. Akkari leg. (ZMUC).

##### Diagnosis.

Most similar to *Ommatoiulus xerophilus* sp. n., but easily distinguished by the shape of promerite and the presence of a distal notch and a small pointed process on solenomerite.

##### Etymology.

Named after the type locality. Châambi Mountain is the highest mountain range in Tunisia, reaching 1550 m at peak Châambi.

##### Description.

Males: L: 17–23 mm, H: 1.6–2 mm, 46–49 PR+1-2 AR+T; females: L: 18.5–32.2 mm, H: 2.4–3.6 mm, 44–50 PR+1-2 AR+T. General colour brownish with a black sputter, dorsally darker, with a black mid-dorsal line. Head dark brown to black with yellow spots in the occipital area, uniformly black frontally, with yellow spots at antennal level and labrum, the latter yellow and brighter; antennae dark brown. Prozonites covered with yellowish-brown spots on a blackish background, also laterally, interrupted by big black spots at ozopore level, dorsally black with a narrow transverse row of yellow spots anteriorly; metazonites glossy pale to whitish; legs light brown to yellowish. Telson: anal valves black, with a yellow sputter, preanal ring black, somewhat paler on the caudal projection; subanal scale light brown. Prozonites with fine oblique striae; metazonites with regular striation, laterally narrower; suture complete, strongly curving at ozopore level; ozopores small, rounded, situated on metazonites, at about their diameter from the suture. Anal valves with numerous submarginal and marginal setae, ca. 2 setae on the surface; subanal scale rounded and setose; preanal ring protruding in a caudal projection with ca. 3+3 setae on the tip and a small hyaline process.

*Male sexual characters*. Mandibular stipites expanded in well-rounded posterior-ventral lobes, first pair of legs hook-shaped, remaining legs with postfemoral and tibial pads.

**Figures 1–5.**
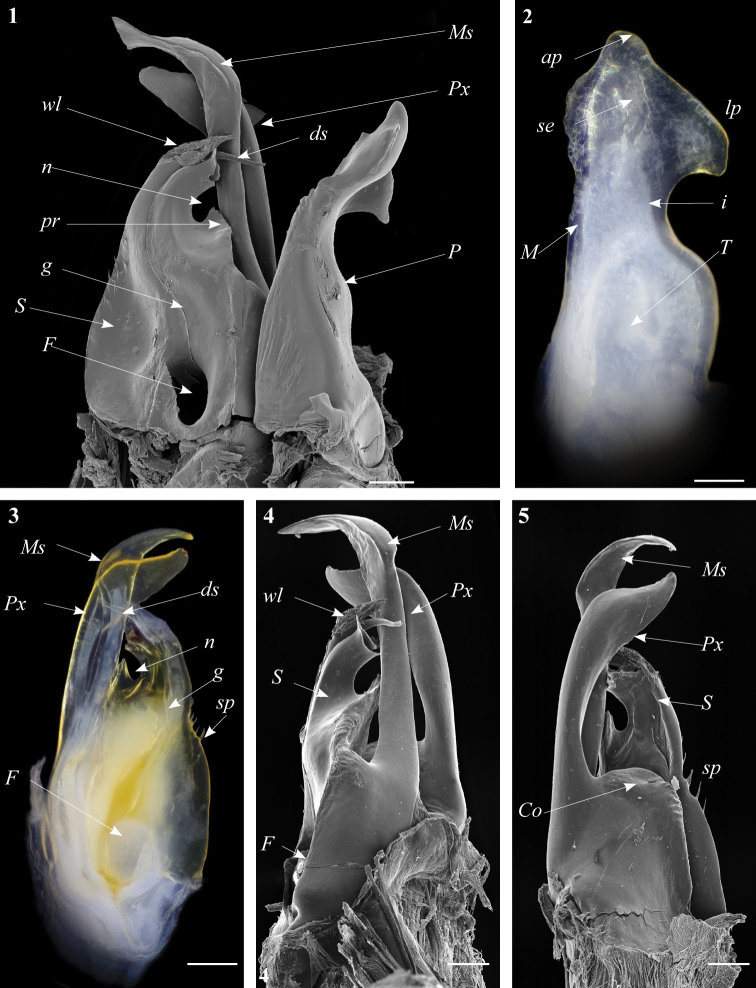
*Ommatoiulus chambiensis* sp. n. paratype, gonopod structures. **1** Right gonopod, mesal view **2** Left promerite, posterior view **3** Left posterior gonopod, mesal view **4** Right posterior gonopod, anterior view **5** Right posterior gonopod, posterior view. Abbreviations: **ap** apical process, **ds** distal process of the solenomerite, **F** fovea, **g** seminal groove, **i** incision on lateral margin of the promerite, **lp** lateral apical process of promerite, **M** mesal ridge, **Ms** mesomerite, **P** promerite, **pr** triangular process of the solenomerite, **Px** paracoxite, **S** solenomerite, **se** serrations, **sp** spikes, **T** remnant of telopodite, **wl** wrinkled lamella. Scale bar: 0.1 mm.

Gonopods. Promerite (**P**) bent anteriad (Figs 1, 2), proximally subrectangular, gradually narrowing in its distal third (Figs 1, 2); lateral margin with a moderately deep rounded incision (**i**). In a posterior view showing a distal process expanded in a subtriangular shape, bearing an apical blunt process (**ap**) and a lateral broad process pointing basad (**lp**); mesal ridge (**M**) apically broadened and delimiting a serrated lateral lamellar process; distal process laterally bearing a strong serrated ridge (**se**) marking a thickness on the posterior surface; telopodite (**T**) remnant ovoid located in the middle of the proximal part.

**Figure 6.**
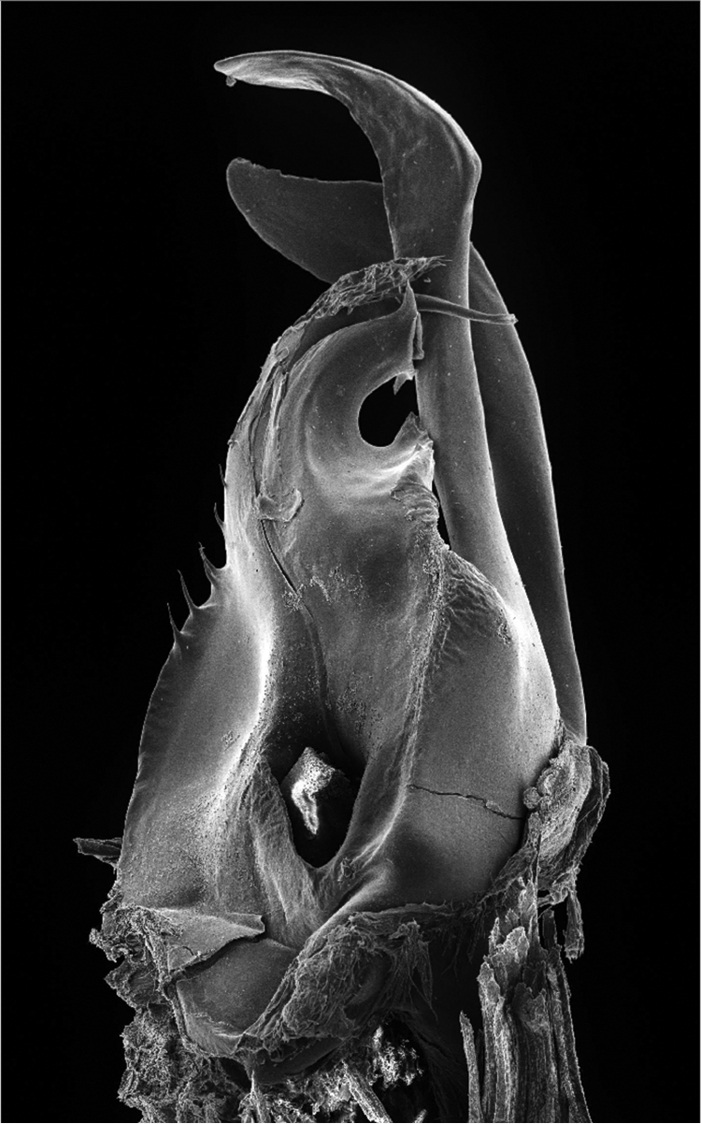
*Ommatoiulus chambiensis* sp. n. paratype,right posterior gonopod. Interactive rotating SEM image. [Morphbank # 831160–831177, 831188]

Posterior gonopod (Figs 3–6): Mesomerite (**Ms)** longer than the other processes of the gonopods, distally curved mesad and narrowing into an apical process folded and tapering toward the apex. Solenomerite (**S**) broad, slightly narrowing at mid-length, proximally with several strong spikes (**sp**) on the posterior margin; anterior margin with a big, serrated process (**pr**) pointing distad, separated from the apical part by a rounded notch (**n**); apical part with an anterior marginally furrowed lamella (Figs 1, 4–6), and a setose wrinkled protruding lamella (**wl**) covering a protruding slender process (**ds**) (Figs 1, 3–6) housing the distal part and the opening of the seminal groove (**g**), the latter running from the fovea (**F**) at the base of the solenomerite up to process **ds**. Paracoxite (**Px**) broad and curved, emerging from a well-rounded coxite (**Co**), distal third broad then gradually narrowing in a rounded apex pointing mesad (Figs 3–6).

##### Distribution.

Known only from the type locality, Châambi Mountain, Arid Bioclimatic zone, central Tunisia.

##### Habitat.

Mixed forest with *Quercus ilex* and *Pinus halepensis*, under stones and in leaf litter.

#### Ommatoiulus crassinigripes

Akkari & Enghoff sp. n.

http://zoobank.org/82B068D7-2ABD-4771-9899-2F2E2C51B9CA

http://species-id.net/wiki/Ommatoiulus_crassinigripes

Figs 7 –13

Schizophyllum punicum : Attems 1903: 144, figs 77–81.
Ommatoiulus punicus : Akkari et al. 2009, in part.

##### Material.

**Holotype**: ♂, W Tunisia, Kasserine Governorate, Châambi National Park, Châambi peak and its surroundings, 35°12.285'N, 8°40.653'E, alt. 1500–1540m, *Quercus ilex*, *Pinus halepensis*, under stones and leaf litter, 9.3.2008, P. Stoev & N. Akkari leg. (ZMUC). **Paratypes**: 4 ♂♂, 2 ♀♀, same data as holotype (ZMUC); 1 ♂, 1 ♀, same data as holotype (NMNHS); 1 ♂, 3 ♀♀, 3 juveniles, CW Tunisia, El Kef, 21.4.1983, Bianchi & Moretti leg. (MSNB); 3 ♂♂, 11 ♀♀, 1 intercalary male, 30 juveniles, CW Tunisia, Makthar, 9.3.1986, Bianchi & Moretti leg. (MSNB); 6 ♀♀, 2 subadults, CW Tunisia, 12 km S Thala, 10.3.1986, Bianchi & Moretti leg. (MSNB); 2 ♂♂, 12 ♀♀, 4 juveniles, C Tunisia, Kairouan Governorate, El Manara, on the road Kairouan-Sidi Bouzid, open and dry area, 35°14'N, 09°45'E, alt. 673m, 17.3.2005, N. Akkari leg. (ZMUC).

##### Diagnosis.

Gonopods resembling those of *Ommatoiulus punicus* and *Ommatoiulus khroumiriensis* sp. n., but differing by the shape of promerite, a much broader and strongly serrated paracoxite, a broader mesomerite bearing subapical serrations on the mesal margin, and the apical processes on solenomerite.

##### Etymology.

An adjunction of Latin words referring to the body size and leg colour, *crassus* meaning fat and *nigripes*, black leg.

##### Description.

Males: L: 24.8–30 mm, H: 2.7–3.6 mm, 45–53 PR+1–2 AR+T. Females: 30–34 mm, H: 3.4–3.9 mm, 45–47 PR+1–2 AR+T. General colour grey, with alternating pale grey and golden brown, darker laterally, with a thin black mid-dorsal line. Head grey, with black sputter frontally, labral zone reddish-brown, brighter at the margin, antennae dark brown. Prozonites pale grey, with big black spots at the level of ozopores and below, a dense black sputter; metazonites whitish anteriorly and golden brown posteriorly, legs black. Telson: anal valves dark grey, bordered with black, preanal ring golden brown, darker on the tip of the caudal projection; subanal scale yellowish. Prozonites with scattered oblique striae; metazonites with regular striation becoming dense laterally; ozopores small and rounded, appearing as brown rounded spots located on metazonites, situated at about their diameter from the suture; the latter complete, only slightly curving at the level of ozopores. Anal valves mostly glabrous at the surface but bearing several submarginal and shorter marginal setae; subanal scale triangular, blunt and setose; preanal ring protruding in a caudal projection, with ca. 2+2 setae and a small hyaline process on the tip.

*Male sexual characters*. Mandibular stipites expanded in well-rounded posterior-ventral lobes, first pair of legs hook-shaped, remaining legs with postfemoral and tibial pads.

**Figures 7–12.**
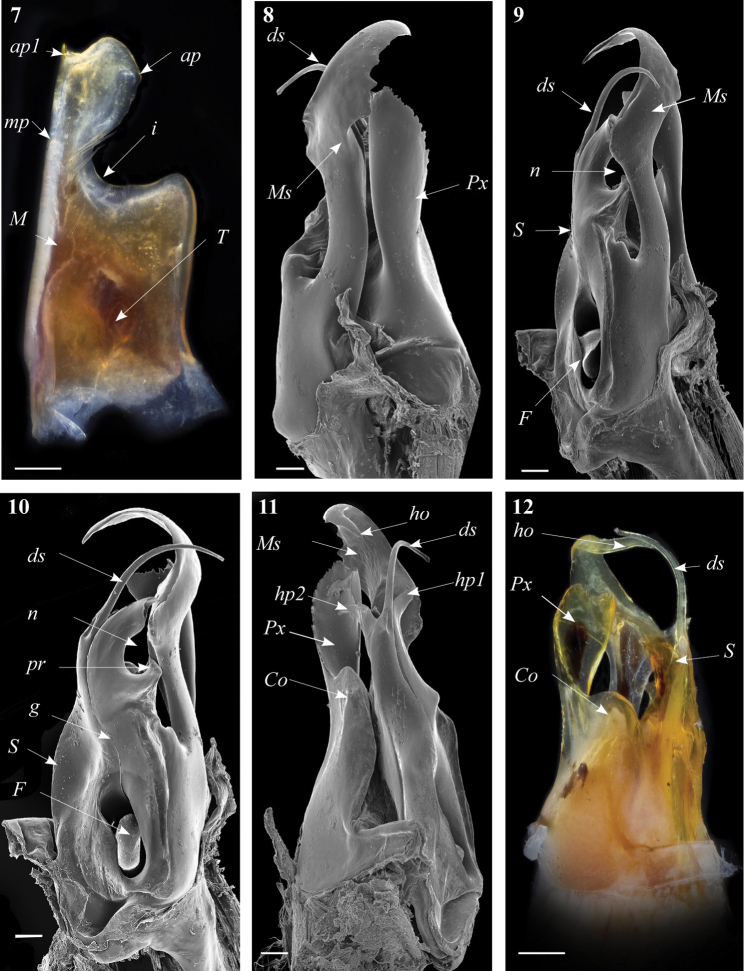
*Ommatoiulus crassinigripes* sp. n. paratype,gonopod structures. **7** Left promerite, posterior view **8** Right posterior gonopod, lateral view **9** Right posterior gonopod, anterior view **10** Right posterior gonopod, mesal view **11** Right posterior gonopod, posterior view **12** Right posterior gonopod latero-posterior view.Abbreviations: **ap** distal process of the promerite, **ap1** apical mesal process, **Co** coxite, **ds** distal process of the solenomerite, **F** fovea, **g** seminal groove, **ho** hook-shaped process, **hp1, hp1** distal processes of the solenomerite, **i** lateral margin incision of the promerite, **M** mesal ridge, **Ms** mesomerite, **mp** distal blunt process of the mesal ridge, **n** notch of the solenomerite, **pr** triangular process of the solenomerite, **Px** paracoxite, **S** solenomerite, **T** remnant of telepodite. Scale bar: 0.1 mm

Gonopods. Promerite (Fig. 7) broad, proximally almost rectilinear, bent 90 degrees at notch level; strongly narrowing in its distal third with a deep incision (**i**) on the lateral margin, latter almost rectilinear; mesal ridge (**M**) broad distally, protruding in a blunt process (**mp**) (Fig. 7); posterior surface irregular on the mesal side, bearing a number of strong setae aligned in front of the notch; distal process (**ap**) laterally broadened and rounded, showing a small mesal serrated process (**ap1**); remnant of telopodite (**T**) as a small bump located proximally.

**Figure 13.**
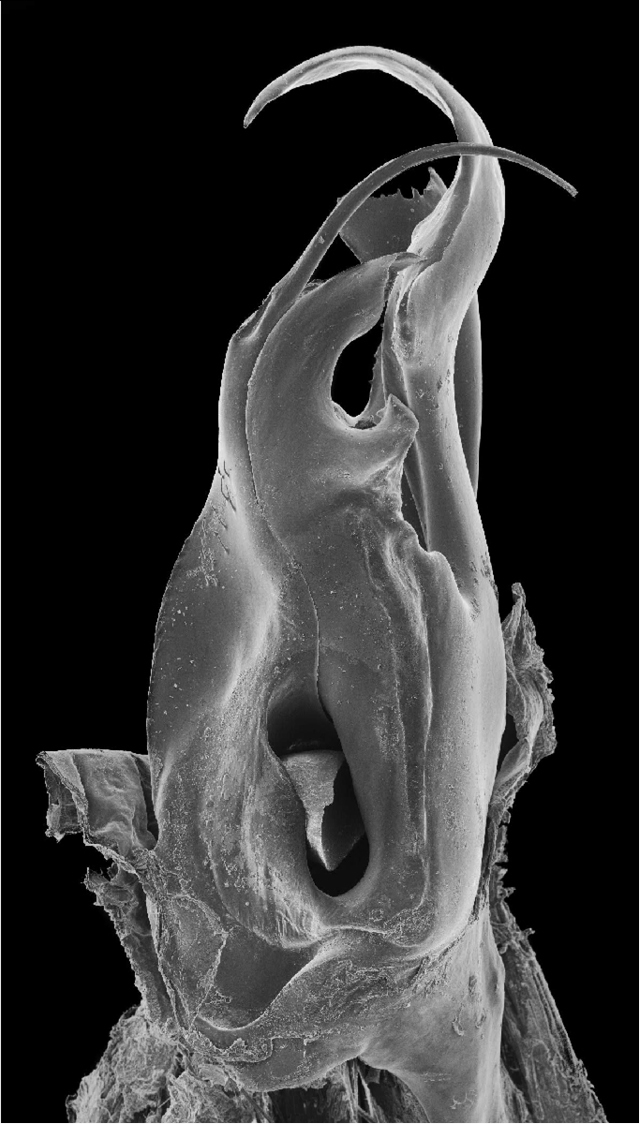
*Ommatoiulus crassinigripes* sp. n. paratype,right posterior gonopod. Interactive rotating SEM image. [Morphbank # 831189–831207]

Posterior gonopod (Figs 8–13). Mesomerite (**Ms**) long, sinuous, distal part asymmetrically enlarged mesolaterally, and showing in lateral view strong serrations at different levels on both margins (Figs 8, 9, 13), gradually narrowed apically in a hook-shaped process (**ho**) curved and tapering toward the apex (Figs 11, 12, 13). Solenomerite (**S**) broadest at the base, narrowing at mid-length and bearing a number of strong setae near the posterior margin (Figs 10, 12, 13); in mesal view showing at mid-length a triangular process (**pr**) pointing distad (Figs 10, 13), latter separated from the apical part by a deep rounded notch (**n**); apically bearing a long curved process (**ds**) pointing mesad, housing the distal part and the opening of the seminal groove (**g**) and emerging between a posterior and an anterior folded hyaline processes (**hp1**, **hp2**) (Figs 11–13). Seminal groove running from the fovea (**F**) at the base of the solenomerite up to process **ds** (Figs 10, 13). Paracoxite (**Px**) lamellar, broad and folded, emerging from a rounded coxite (**Co**), distally broadened, apical margin almost horizontal, and together with the posterior margin showing many strong, short serrations (Figs 8, 11–13).

##### Distribution.

Semi-arid and Arid bioclimatic zones in west central Tunisia, recorded from the governorates Kasserine, El Kef, Thala and Kairouan.

##### Habitat.

Dry and open habitats, to 1500 m in Châambi Mountain.

#### Ommatoiulus kefi

Akkari & Enghoff sp. n.

http://zoobank.org/93E41C97-AA12-45D5-BD86-F2B0ACF02465

http://species-id.net/wiki/Ommatoiulus_kefi

Figs 14 –19

##### Material.

**Holotype**: ♂, W Tunisia, El Kef Governorate, 13 km S El Kef, 22.iv.1981, Bianchi & Moretti leg. (MSNB).

##### Diagnosis.

Differing from all congeners by having a tri-lobate distal part of promerite and a bifurcated apical part of mesomerite, the latter divided in two short oppositely directed processes.

##### Etymology.

Named after El Kef city, the type locality of the species.

##### Description.

Male: L: 26 mm, H: 2.7 mm, 53 PR+1 AR+T. General colour alternating whitish and blackish with a thin black mid-dorsal line. Head brown, lighter on the frontal part, with yellowish spots at antennal level, labral zone yellowish, becoming brighter at the margin, antennae brownish. Prozonites pale grey, dorsally scarcely sputtered with black; metazonites anteriorly dark, with a blackish background and a line of light brown spots below ozopores; legs whitish. Telson: anal valves and preanal ring blackish, paler towards caudal projection, subanal scale yellowish. Prozonites with scattered oblique striae; metazonites with regular striation becoming dense laterally; suture complete and rectilinear; ozopores small, rounded and located in metazonites, well apart from the suture. Anal valves with numerous submarginal and marginal setae and ca. 1-2 setae on the surface; subanal scale triangular, blunt and setose; preanal ring protruding in a caudal projection, with ca. 3+3 setae and a small hyaline process on the tip.

*Male sexual characters*. Mandibular stipites expanded in rounded posterior-ventral lobes, first pair of legs hook-shaped, remaining legs with postfemoral and tibial pads.

**Figures 14–18.**
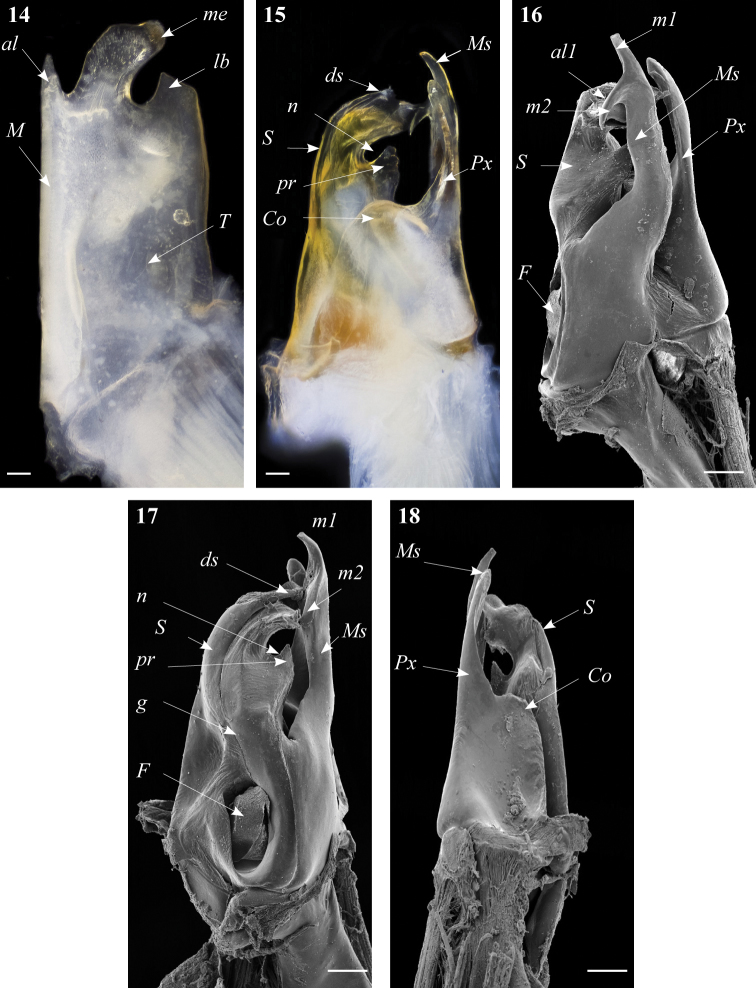
*Ommatoiulus kefi* sp. n. holotype, gonopod structures. **14** Left promerite, posterior view **15** Left posterior gonopod, posterior view **16** Right posterior gonopod, anterior view **17** Right posterior gonopod, mesal view **18** Right posterior gonopod, mesal view. Abbreviations: **al** apical lobe of the promerite, **al1** apical folded lamella of the solenomerite, **Co** coxite, **ds** distal process of the solenomerite, **F** fovea, **g** seminal groove, **lb** lateral lobe, **m1, m2** apical processes of the mesomerite, **M** mesal ridge, **me** median lobe, **Ms** mesomerite, **n** notch of the solenomerite, **pr** triangular process of the solenomerite, **Px** paracoxite, **S** solenomerite, **T** telopodite. Scale bar: 0.1 mm

Gonopods. Promerite (Fig. 14) in posterior view subrectangular, mesal ridge (**M**) fairly broad, distally narrowing and protruding into a pointed apical lobe (**al**); apical margin protruding in a curved median lobe (**me**) pointing laterad and a shorter broad lateral lobe (**lb**); the three apical lobes separated by two rounded incisions; lateral margin almost rectilinear. Remnant of telopodite (**T**) ovoid, located at mid-length of promerite.

**Figure 19.**
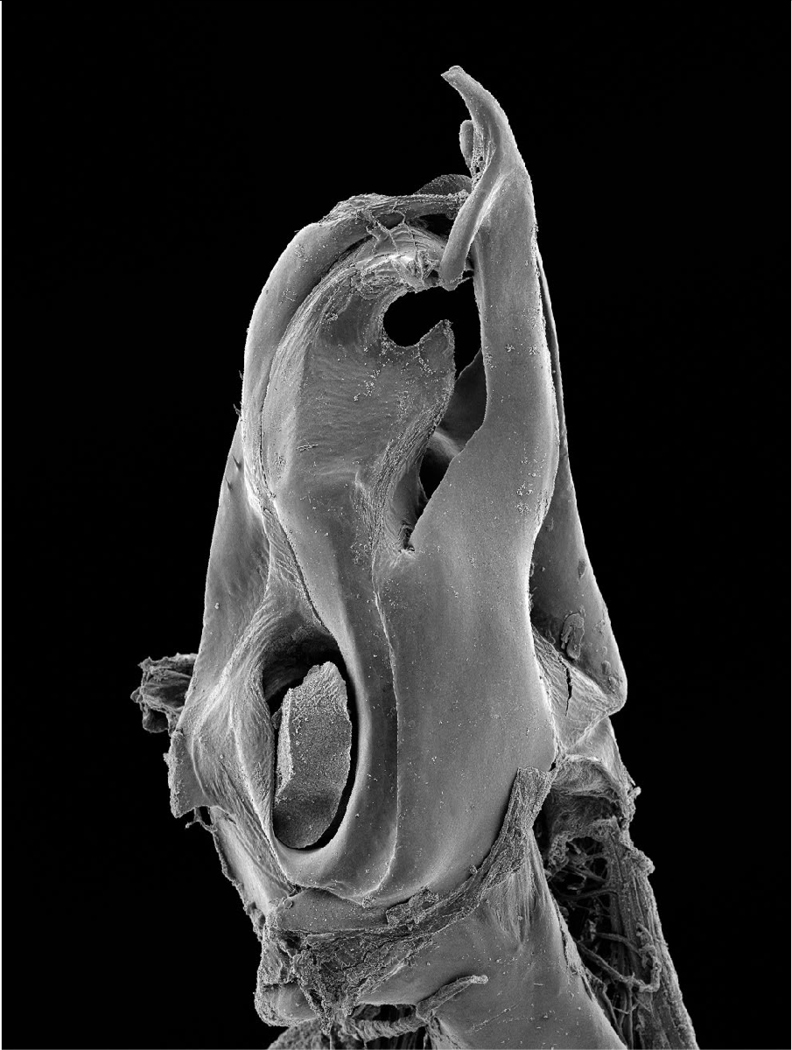
*Ommatoiulus kefi* sp. n. holotype, right posterior gonopod. Interactive SEM image. [Morphbank # 831208-831227]

Posterior gonopod (Figs 15–19): Mesomerite (**Ms**) broadest at the base, distally protruding in a uniformly broad process, apically splitting into two short and curved processes, pointing in opposite directions (**m1**, **m2**) (Figs 16, 17, 19); solenomerite (**S**) broad at the base, slightly narrowing at mid-length and showing a triangular process (**pr**) separated from the rest of the processes by a rounded (**n**) (Figs 17, 19), apical part of the solenomerite complex with a broad lamella (**al1**) extended latero-mesad, downturned and marginally furrowed (Fig. 16). Seminal groove (**g**) running from the fovea (**F**) at the base of solenomerite up to a slender and short conical process (**ds**) emerging on top of the median part of the apical lamella and pointing anteriad (Figs 15, 17). Paracoxite (**Px**) stout, with smooth margins, emerging from a broad rounded coxite (**Co**) (Figs 18, 19).

##### Distribution.

Semi-arid bioclimatic zone in western Tunisia; hitherto known only from the type locality near El Kef city.

##### Comments.

We have examined three females (MSNB) collected from the same locality but could not assign them with certainty to *Ommatoiulus kefi* as they show a different colour pattern.

#### Ommatoiulus khroumiriensis

Akkari & Enghoff sp. n.

http://zoobank.org/05BAED95-725C-4395-92C3-6BF22A01CE18

http://species-id.net/wiki/Ommatoiulus_khroumiriensis

Figs 20 –26

Archiulus punicus : Attems (1926): 191, figs 240, 241.
Ommatoiulus punicus : Akkari et al. 2009, in part.
Ommatoiulus cf. *punicus*: Enghoff et al. 2011: 610.

##### Material.

**Holotype**: ♂, NW Tunisia, Jendouba Governorate, Aïn Draham, Col des Ruines, 1.11.2009, N. Akkari leg. (ZMUC). **Paratypes**: 2 ♂♂, 5 ♀♀, NW Tunisia, Jendouba Governorate, Aïn Draham, Col des Ruines, 1.11.2009, N. Akkari leg. (ZMUC); 2 ♂♂, 6 ♀♀, NW Tunisia, Jendouba Governorate, 7 km south Aïn Draham, les chênes, 22.3.1986, ZMUC expedition; 1 ♂, 1 ♀, 4 immatures, 5-18.3.1988, NW Tunisia, Jendouba Governorate, Aïn Draham area, ZMUC expedition; 1 ♂, 1 ♀, 1 intercalary male, NW Tunisia, Jendouba Governorate, Aïn Draham, 19.11.2003, forest with *Quercus suber* and *Quercus faginea*, under stones, N. Akkari leg. (NMNHS); 2 ♂♂, 5 ♀♀, 2 juveniles, NW Tunisia, Jendouba Governorate, Hammam Bourguiba, 36°45'N, 08°35'E, alt. 158m, mixed forest with *Pinus pinaster* and *Quercus suber*, under stones, 31.10.2009, N. Akkari leg. (ZMUC); 3 ♂♂, NW Tunisia, Jendouba Governorate, Aïn Draham, 36°47'N, 8°41'E, alt. 511m, 3.10.2005, N. Akkari leg. (ZMUC); 1 ♂, 2 ♀♀, NW Tunisia, Jendouba Governorate, Aïn Draham, 36°47'N, 8°41'E, 760m, *Quercus suber*-*Erica* forest, 11.3.2009, N. Akkari & H. Enghoff leg. (ZMUC); 3 ♂♂ Jendouba Governorate, route Aïn Draham- Fernana, 36°43'N, 8°40'E, *Quercus suber*-*Erica* forest, 9.3.2009, N. Akkari & H. Enghoff leg. (ZMUC); 1 ♂, 2 ♀♀, Jendouba Governorate, route Aïn Draham- Béni M’tir, 36°43'N, 8°42'E, *Quercus suber*-*Erica* forest, 10.3.2009, N. Akkari & H. Enghoff leg. (ZMUC).

##### Diagnosis.

Similar to *Ommatoiulus punicus* and *Ommatoiulus crassinigripes* sp. n. but readily distinguished by the shape of promerite having a deeper notch extended basad, much slenderer processes of posterior gonopods, and a more sinuous mesomerite devoid of conspicuous serrations.

##### Etymology.

The species name refers to the natural region of Khroumirie, NW Tunisia, to which the species seems confined.

##### Description.

Males: L: 26–27 mm, H: 2–2.8 mm, 43–48 PR+1–2 AR+T, females: L: 30–37 mm, H: 4–4.3, 44–48 PR+1 AR+T. General colour dark grey, alternating with brown-yellow laterally, and with a thin black mid-dorsal line. Head dark reddish-brown; occipital area blackish, with brown-reddish- spots; frontal part uniformly black, labral zone brown-reddish- to yellowish at margin, antennae brownish. Prozonites uniformly grey, with a pale narrow stripe anteriorly; metazonites darker, brown-greyish, densely sputtered with black, colour gradually vanishing on the sides, below ozopore level yellow-brownish; legs light brown. Telson: anal valves black, preanal ring blackish, caudal projection brown-reddish, subanal scale light brown to yellowish.

Prozonites with scattered oblique striae; metazonites densely striated; suture complete, curving at ozopore level; ozopores small, rounded and located on metazonites, situated at about their diameter from the suture. Anal valves with 4-5 setae on the surface, a submarginal row of 12-13 setae and numerous short marginal ones; subanal scale triangular and setose; preanal ring protruding in a caudal projection with ca. 3+3 setae and a small hyaline process on the tip.

*Male sexual characters*. Mandibular stipites expanded in rounded posterior-ventral lobes, first pair of legs hook-shaped, remaining legs with postfemoral and tibial pads.

**Figures 20–25.**
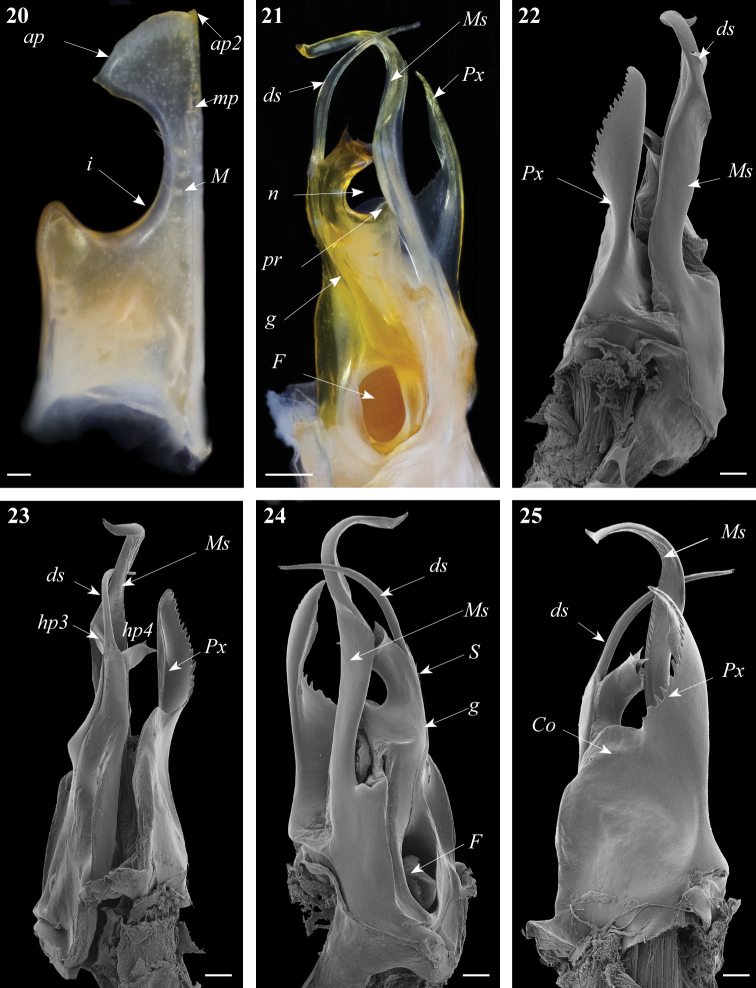
*Ommatoiulus khroumiriensis* sp. n. paratypes, gonopod structures. **20** Right promerite, posterior view **21** Right posterior gonopod, mesal view **22** Right posterior gonopod, lateral view **23** Right posterior gonopod, meso-posterior view **24** Right posterior gonopod, anterior-mesal view **25** Right posterior gonopod,anterior view. Abbreviations: **ap** distal process of the promerite, **ap2** lobed process, **Co** coxite, **ds** distal process of the solenomerite, **F** fovea, **g** seminal groove, **hp3, hp4** distal processes of the solenomerite, **i** lateral incision of the promerite, **mp** blunt mesal process, **M** mesal ridge, **Ms** mesomerite, **n** notch of the solenomerite, **pr** triangular subapical process of the solenomerite, **Px** paracoxite, **S** solenomerite. Scale bar: 0.1 mm.

Gonopods. Promerite (Fig. 20) strongly narrowed distally with a deep lateral incision (**i**) extending meso-basad, distal process (**ap**) broad, subtriangular, with two pointed edges, the tip of apical process with a small pointed lobe (**ap2**); mesal ridge (**M**) distally protruding in a blunt small cylindrical process (**mp**), posterior surface of promerite with a row of strong setae emerging at the level of the notch, in close proximity to the mesal ridge.

**Figure 26.**
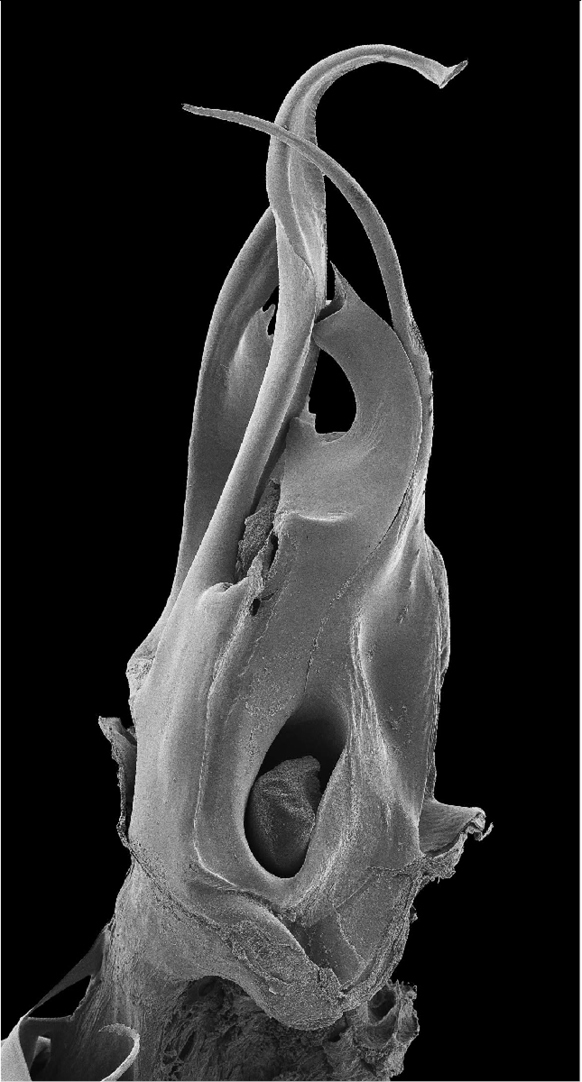
*Ommatoiulus khroumiriensis* sp. n. paratype, left posterior gonopod. Interactive SEM image. [Morphbank # 831016–831034]

Posterior gonopod (Figs 21–26). Mesomerite (**Ms**) large, longer than the other processes, uniformly broad, sinuous; distal third constricted to less than half breadth and apically protruding into a slender curved process, latter tapering and pointing mesad (Figs 21, 23, 24, 26). Solenomerite (**S**) broadest at the base, narrowing at mid-length, and bearing a number of strong setae near the posterior margin, distally with a broad, blunt triangular process (**pr**) separated from the apical part by a rounded notch (**n**), and with a long curved process (**ds**) protruding between two apical hyaline processes (**hp3, hp4**) and housing the apical part of seminal groove (**g**), the latter (**g**) running from the fovea (**F**) located at the base of the solenomerite (**S**) up to process **ds**.Paracoxite (**Px**) emerging from a depressed coxite (**Co**); **Px** curved, half as broad as in *Ommatoiulus crassinigripes*, gradually narrowing distad; lateral and apical margins, with a saw-like strongly jagged margin (Figs 22–26).

##### Distribution.

Humid bioclimatic zone in northwestern Tunisia; known from Aïn Draham, Fernana and Hammam Bourguiba in Khroumirie, Jendouba Governorate.

##### Habitat.

Mixed forests dominated by *Quercus faginea* and *Quercus suber*, or *Pinus pinaster* and *Quercus suber*.

#### Ommatoiulus xenos

Akkari & Enghoff sp. n.

http://zoobank.org/09D98AF5-9AD3-41E0-8B54-624E31B72480

http://species-id.net/wiki/Ommatoiulus_xenos

Figs 27–30

##### Material.

**Holotype**: ♂, Tunisia (“Tunis”), 1861, J.P. Coindé leg. (MNHN). **Paratypes**: 5 ♀♀, Tunisia (“Tunis”), 1861, J.P. Coindé leg. (MNHN).

##### Diagnosis.

Resembling *Ommatoiulus chambiensis* and *Ommatoiulus xerophilus* spp. n. in size and general shape of gonopods, but distinguished by the shape of promerite, a much more slender mesomerite and shorter and stouter paracoxite.

##### Etymology.

The species name is a Greek noun meaning ‘stranger’, emphasising the fact that this species, found surprisingly in the collection of the MNHN shortly before completion of the manuscript, had remained unknown and out of the sight of a number of myriapodologists for more than 150 years.

##### Description.

Male: L: 20.5 mm, H: 1.85 mm, 47 PR+2 AR+T; females: L: 18.5–21 mm, H: 2.26–2.46 mm, 42–48 PR+2–3 AR+T. General colour faded, generally grey-greenish (very likely an artefact from the decomposition of the inserted label), somewhat lighter laterally. Head pale in the occipital and labral areas; antennae and legs darker. Prozonites with darker triangular spots laterally, latter situated along the ozopores line and forming two longitudinal dark bands, dorsally separated by a pale one; dorsum crossed by dark triangular spots and showing a thin black mid-dorsal line; metazonites mostly pale and glossy. Telson: anal valves and preanal ring dark, subanal scale pale.

Prozonites with fine striae; metazonites with regular striae, denser on the sides, suture complete, curving at the level of ozopores; ozopores small, rounded, situated on metazonites situated at about their diameter from the suture. Anal valves setose; preanal ring with 3-4 setae on each lateral side, protruding in a short caudal projection with 1-4/5 setae and a small hyaline process on the tip. Subanal scale blunt to rounded and setose.

*Male sexual characters*. Mandibular stipites expanded in rounded posterior-ventral lobes, first pair of legs hook-shaped, remaining legs with postfemoral and tibial pads.

**Figures 27–30.**
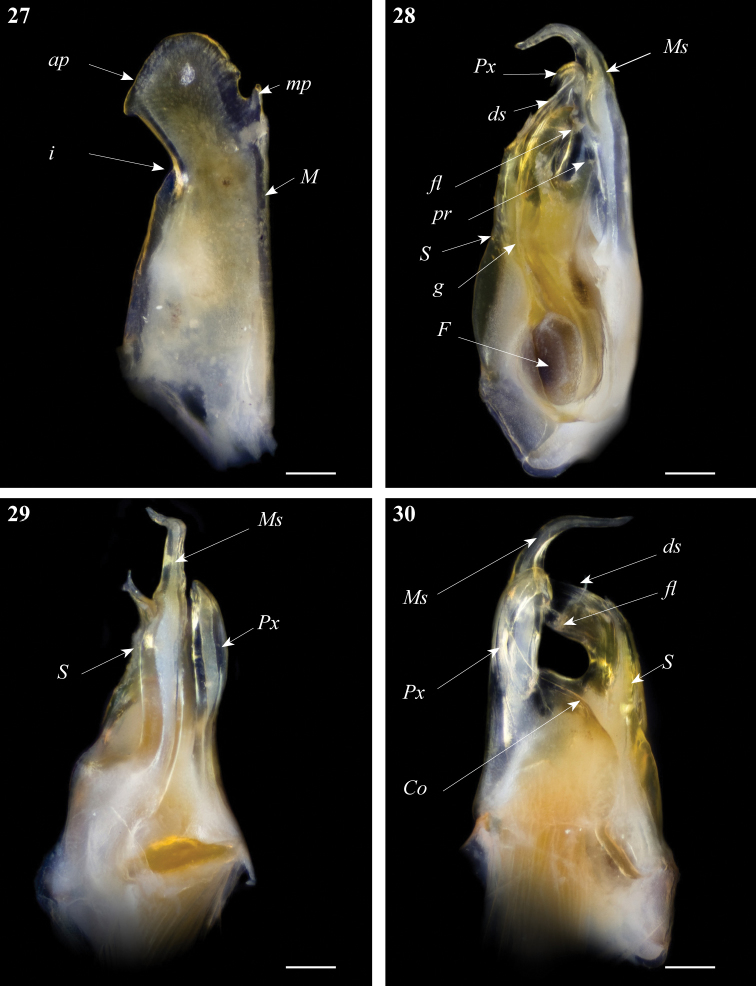
*Ommatoiulus xenos* sp. n. holotype,gonopod structures. **27** Right promerite, posterior view **28** Right posterior gonopod, mesal view **29** Right posterior gonopod, antero-lateral view **30** Right posterior gonopod, posterior view. Abbreviations: **Co** coxite, **ds** distal process of the solenomerite, **F** fovea, **fl** folded lamella, **g** seminal groove, **i** lateral incision of the promerite, **M** mesal ridge, **mp** distal process, **Ms** mesomerite, **pr** blunt process of the solenomerite, **Px** paracoxite, **S** solenomerite. Scale bar: 0.1 mm.

Gonopods. Promerite (Fig. 27) gradually narrowed distally, lateral margin with a shallow incision (**i**); apical process of promerite with a rounded margin pointing laterad; mesal ridge (**M**) narrow, distally protruding in a pointed apex (**mp**) separated from the apical process by a small apical incision; remnant of telopodite not very conspicuous.

Posterior gonopod (Figs 28–30): Mesomerite (**Ms**) uniformly broad proximally, strongly narrowed in its distal third and bent posteriad (Figs 28, 29); solenomerite (**S**) broad, with scattered setae on posterior margin, narrowing at mid-length, and bearing a large blunt process (**pr**); solenomerite apically with a broad folded lamella (**Fl**) and a small wrinkled lamella laying on the top of a slender and slightly protruding process (**ds)** housing the distal part of the seminal groove (**g**); seminal groove running from the fovea (**F**) and opening at the apex of process **ds** (Fig. 28). Paracoxite (**Px**) stout, distally curved mesad and narrowed into a slender apex pointing basad emerging from a broad and rounded coxite (**Co**) (Fig. 30).

##### Distribution.

Exact locality unknown. The label mentions ‘Tunis’ which presumably refers to Tunisia in general.

##### Habitat.

Unknown.

##### Remarks.

The collector of this species, J.P. Coindé, who was a ‘zoologist-traveler’, made a collecting trip to Tunisia in 1861 during which he visited several localities throughout the country. Although we are certain that *Ommatoiulus xenos* sp. n., found by chance in an obscure jar among several unidentified myriapods from North Africa, labelled ‘Brolemann unidentified’, was collected in Tunisia, we couldn’t determine with certainty the locality where this species was collected 152 years ago.

#### Ommatoiulus xerophilus

Akkari & Enghoff sp. n.

http://zoobank.org/6C3A27BC-A781-4EF3-B2BD-2275C59126FF

http://species-id.net/wiki/Ommatoiulus_xerophilus

Figs 31 –35

##### Material.

**Holotype**: ♂, W Tunisia, Kasserine Governorate, Châambi National Park, surroundings of the park´s guest house, 35°10.139'N, 8°40.486'E, alt. 950–1000m, scarce trees, *Pinus halepensis*, *Thuja*, under stones, logs and in leaf litter, 8.3.2008, P. Stoev & N. Akkari leg. (ZMUC). **Paratypes**: 1 ♂, 2 ♀♀, W Tunisia, Kasserine Governorate, Châambi National Park, surroundings of the park's guest house, 35°10.139'N, 8°40.486'E, alt. 950m, scarce trees, *Pinus halepensis*, under stones, 7.3. 2008, N. Akkari & P. Stoev leg. (ZMUC); 2 ♂♂, 12 ♀♀, 8 subadult ♀♀, 8 juveniles, W Tunisia, El Kasserine Governorate, Châambi National Park, surroundings of the park’s guest house, 35°10. 139'N, 8°40.486'E, alt. 950–100m, scarce trees, *Pinus halepensis*, *Thuja*, under stones, logs and in leaf litter, 8.3.2008, P. Stoev & N. Akkari leg. (ZMUC); 1 ♂, 2 ♀♀, same data (NMNHS).

##### Diagnosis.

Resembling *Ommatoiulus chambiensis* in the structure of mesomerite, paracoxite, but well distinguished from the latter by the characteristic globular apex of promerite and the shape of solenomerite devoid of a rounded notch.

##### Etymology.

The species name is a Greek adjective referring to the affinity of the species for dry habitats.

##### Description.

Males: L: 15.7–15.9 mm, H: 1.54–1.65 mm, 44–46 PR+1–2 AR+T; females: L: 10.2–23.1 mm, H: 1.44–2.56 mm, 39–46 PR+1–4 AR+T. General colour black to brownish, light brown on the sides; dorsum pale yellow, crossed by thick black mid-dorsal spots. Head dark to blackish with yellow spots in the occipital area, frontal part uniformly black showing yellow spots at antennal level, labral zone yellowish, brighter at margin; antennae brownish. Prozonites blackish with light brown-yellowish spots becoming dominant laterally, just below the ozopore line; dorsally pale, with big, irregularly shaped black spots; metazonites predominantly grey-whitish and glossy, legs yellowish. Telson: anal valves dark brown-blackish, with dense yellow sputter, preanal ring blackish sputtered with yellow, dorsal side and caudal projection mostly pale, subanal scale yellowish.

Prozonites with fine striae; metazonites with regular striae, becoming denser laterally, suture complete, curving at the level of ozopores; latter small, rounded, situated on metazonites situated at about their diameter from the suture. Anal valves setose, with 6-7 setae on the surface and numerous submarginal and marginal setae; subanal scale rounded and setose; preanal ring with 1+1 setae on the sides, protruding in a caudal projection with 3+3 setae and a small hyaline process on the tip.

*Male sexual characters*. Mandibular stipites expanded in rounded posterior-ventral lobes, first pair of legs hook-shaped, remaining legs with postfemoral and tibial pads.

**Figures 31–34.**
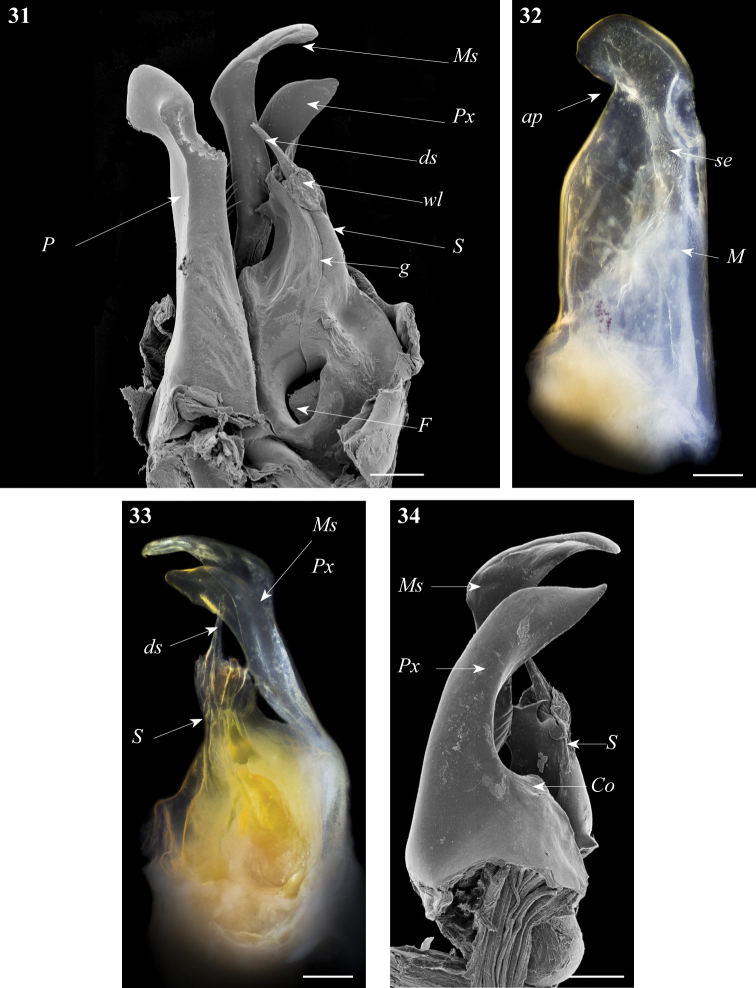
*Ommatoiulus xerophilus* sp. n. paratype,gonopod structures. **31** Left gonopod, mesal view **32** Rightpromerite, posterior view **33** Rightposterior gonopod, mesal view **34** Rightposterior gonopod, latero-posterior view. Abbreviations: **ap** distal process of the promerite, **Co** coxite, **ds** distal process of the solenomerite, **F** fovea, **g** seminal groove, **M** mesal ridge, **Ms** mesomerite, **P** promerite, **Px** paracoxite, **S** solenomerite, **se** serrations, **wl** wrinkled lamella. Scale bar: 0.1 mm.

Gonopods. Promerite (**P**) (Figs 31, 32) bent anteriad, not very broad, with parallel margins, mesal ridge (**M**) broad, distally narrowing and truncate, bearing several serrations (**se**) and separated from the apical lobe (**ap**); by an incision; lateral margin mostly rectilinear, only slightly narrowing subapically at the level of the mesal incision; apical lobe globular, with rounded margin, curved laterad; posterior surface with a number of strong setae aligned distally; telopodite remnant inconspicuous.

**Figure 35.**
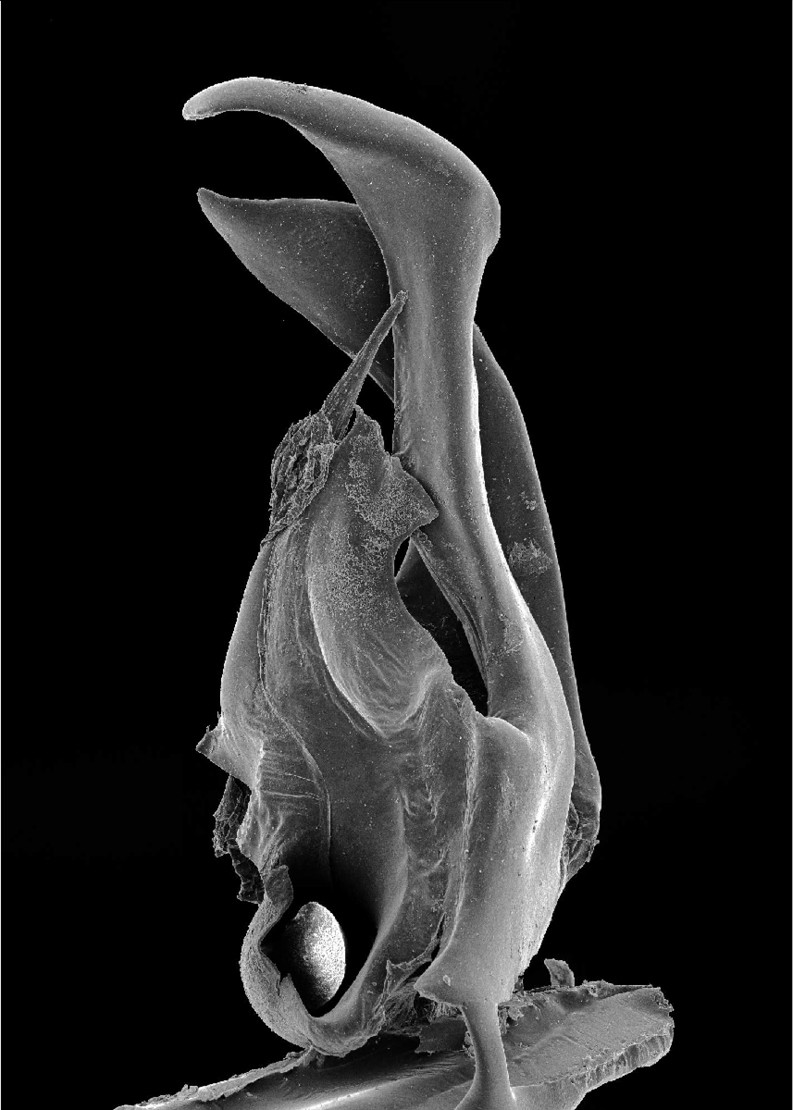
*Ommatoiulus xerophilus* sp. n. paratype, right posterior gonopod. Interactive SEM image. [Morphbank # 831246–831263]

Posterior gonopod (Figs 31, 33–35): Mesomerite (**Ms**) similar to *Ommatoiulus chambiensis* but broader, strongly truncated and distally bent mesad (Figs 33–35); solenomerite (**S**) broad, with scattered setae on posterior margin, strongly narrowing at mid-length, apical part with a wrinkled blunt lamella (**wl**) covering a slender and protruding process (**ds)** housing the distal part of the seminal groove (**g**) (Figs 31, 33, 35); the latter running from the fovea (**F**) (Figs 31, 33–35) up to process **ds**. Paracoxite (**Px**) broad, curved mesad, emerging from a depressed coxite (**Co**), distal third slightly enlarged, apically narrowing into a pointed apex, directed mesad (Figs 33–35).

##### Distribution.

Arid bioclimatic zone, central Tunisia; hitherto known only from the type locality, Châambi Mountain.

##### Habitat.

Open areas with scattered *Pinus halepensis* trees.

#### Ommatoiulus zaghouani

Akkari & Enghoff sp. n.

http://zoobank.org/F23E46BA-1E8C-4B6D-BF46-73D9A37E8E42

http://species-id.net/wiki/Ommatoiulus_zaghouani

Figs 36–39

##### Material.

**Holotype**: ♂, NE Tunisia, Zaghouan Governorate, Jebel Zaghouan, 36°23'N, 10°06E, alt. 365m, *Pinus* forest, 13.3.2009, N. Akkari & H. Enghoff leg. (ZMUC).

##### Diagnosis.

Gonopods resembling those of *Ommatoiulus seurati* but distinguished by a broader distal part of the promerite, a subapical lateral projection on the mesomerite and a much shorter solenomerite.

##### Etymology.

Named after Jebel Zaghouan, the type locality.

##### Description.

Male: L: 28.5 mm, H: 2.56 mm, 49 PR+1 AR+T. General colour alternate dark and light golden brown; dorsum with a thin black axial line. Head dark to blackish, with brownish spots on the frontal part and on the mandibular stipites, labral zone and mouth parts pale, marginally bright yellow; antennae brownish. Prozonites dark to blackish, covered with yellowish spots; metazonites pale brown to whitish laterally and golden brown dorsally, legs yellowish. Telson: anal valves black, preanal ring blackish, caudal projection yellowish, subanal scale yellowish.

Prozonites with fine irregular striae; metazonites with regular striae, becoming dense laterally, suture complete, curving at the level of ozopores; ozopores small, rounded, situated in metazonites, situated at about their diameter from the suture. Anal valves setose, with 4-6 setae on the surface, ca 14 submarginal and numerous marginal setae; subanal scale rounded and setose; preanal ring with 2+2 setae on the sides, protruding in a caudal projection with (6-7)+(6-7) setae on the tip and bearing a small hyaline process.

*Male sexual characters*. Mandibular stipites expanded in rounded posterior-ventral lobes, first pair of legs hook-shaped, remaining legs with postfemoral and tibial pads.

**Figures 36–39.**
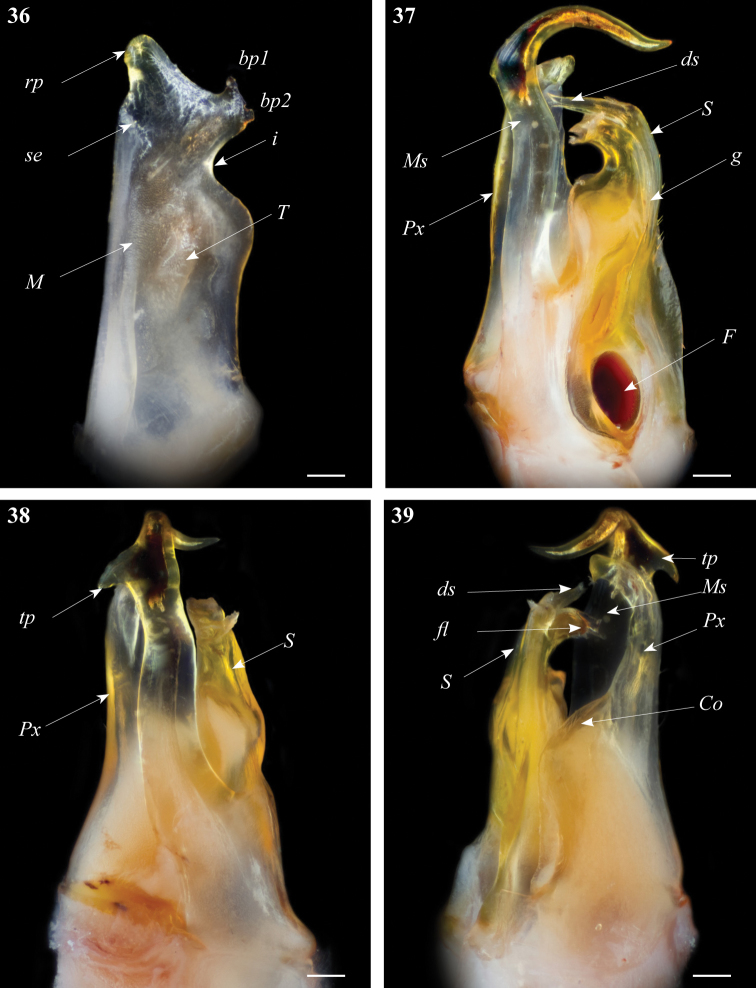
*Ommatoiulus zaghouani* sp. n. holotype, gonopod structures. **36** Left promerite, posterior view **37** Left posterior gonopod, mesal view **38** Left posterior gonopods, anterior view **39** Left posterior gonopods, postero-lateral view. Abbreviations: **bp1, bp2**: small bumps on the apical lateral process, **Co** coxite, **ds** distal process of the solenomerite, **F** fovea, **fl** folded lamella, **g** seminal groove, **M** mesal ridge, **Ms** mesomerite, **i** lateral incision on the promerite, **Px** paracoxite, **rp** apical mesal process of the promerite, **S** solenomerite, **se** serrated process, **tp**: triangular distal process. Scale bar: 0.1 mm.

Gonopods. Promerite (Fig. 36) proximally subrectangular, strongly narrowed distally by a deep incision (**i**) on the lateral margin; mesal ridge (**M**) broad, distally protruding in a serrated edge (**se**); apical part with a mesal triangular blunt process (**rp**) protruding mesodistad, and a lateral protruding process with two small apical bumps (**bp1**, **bp2**); posterior surface of promerite showing few scattered setae near the mesal margin; remnant of telopodite (**T**) large and ovoid, located at mid-length of the process.

Posterior gonopod (Figs 37–39): Mesomerite (**Ms**) large, and uniformly broad (Figs 37, 38) with a distal triangular pointed extension on the lateral margin (**tp**), distal third strongly curved mesoposteriad and narrowed in a long and slender apical process (Figs 37, 38, 39); solenomerite (**S**) broad, with scattered setae on posterior margin; anteriorly simply rounded devoid of processes; apically with a hyaline folded lamella (**fl**) and a slightly protruding process (**ds)** housing the distal part of the seminal groove (**g**); the latter running from the fovea (**F**) (Fig. 37) up to process **ds**. Paracoxite (**Px**) stout and curved apically slightly narrowing into a rounded apex directed mesad, coxite broad (**Co**) (Fig. 39).

##### Distribution.

Semi-arid bioclimatic zone in northeastern Tunisia; known only from Zaghouan Mountain.

##### Habitat.

Forest dominated by *Pinus halepensis*.

### Updates to the list of *Ommatoiulus* species in North Africa

Akkari et al. (2009) summarized all records of julidan millipedes, including *Ommatoiulus* species, from Tunisia and provided a complete list of the North African members of the order. A number of additions and corrections to the lists are presented here:

- We refute the presence of *Ommatoiulus diplurus appendiculatus* (Brolemann, 1925) in Algeria. This taxon is based on females and juveniles only, and Akkari and Enghoff (2012) already regarded the presence of *Ommatoiulus diplurus* (Attems, 1903) in North Africa uncertain.
- The record of *Ommatoiulus aumalensis* (Brolemann, 1925) from Tunisia by Akkari et al. (2009) was due to a misidentification and actually refers to *Ommatoiulus fuscounilineatus* (Lucas, 1846).
- *Ommatoiulus sempervirilis* Akkari & Enghoff, 2011 was described from the Tunisian islands Galita and Zembretta (Akkari and Enghoff 2011).
- *Ommatoiulua chambiensis*, *Ommatoiulus crassinigripes*, *Ommatoiulus kefi*, *Ommatoiulus khroumiriensis*, *Ommatoiulus xerophilus*, *Ommatoiulus xenos* and *Ommatoiulus zaghouani* spp. n., are described from northern and central Tunisia in the present paper bringing the overall number of *Ommatoiulus* species in Tunisia to 12, and to 28 in North Africa.
- We refute the presence of *Ommatoiulus lapidarius* (Lucas, 1846) in Libya. This record was based on the synonymy of *Julus rimosus* Karsch 1881 (see Manfredi 1939, Schubart 1952) with *Ommatoiulus lapidarius*, but re-examination of the holotype of *Julus rimosus* (Museum für Naturkunde, Berlin) has shown that this is a valid species (Akkari 2013).

## Discussion

**Figure 40.**
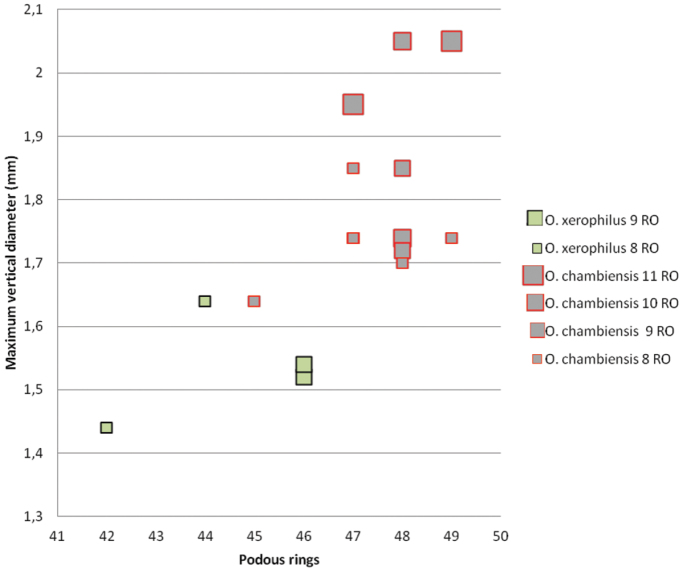
*Ommatoiulus chambiensis* and *Ommatoiulus xerophilus* spp. n.Scatter diagram illustrating size of adult males expressed as maximum vertical body diameter (mm) vs number of podous rings in different developmental stadia (8–11RO).

Three of the new Tunisian *Ommatoiulus* species were found in Châambi Mountain, which is the highest mountain range in Tunisia. The mountain is located only a few kilometres from the Algerian border and in spite of its arid character is known to harbour a number of endemic species (cf. Kovařík 2006, Hartenberger 1986), which is here confirmed by the finding of *Ommatoiulus chambiensis*, *Ommatoiulus xerophilus* and *Ommatoiulus crassinigripes* spp. n. in semi-arid open habitats of the mountain.With regard to morphology, *Ommatoiulus chambiensis* and *Ommatoiulus xerophilus* show great resemblance in the gonopod structure as they both have apical serrations connecting the mesal ridge of the promerite with the apical processes of the promerite, which is reminiscent of similar structures found in the *Ommatoiulus fuscounilineatus* species group (see Akkari and Enghoff 2012, figs 94–96). The posterior gonopods of both species are outstanding in having a large, distally curved mesomerite and paracoxite, and a relatively short and simply structured solenomerite. However, clear differences between them were observed in the apex of the promerite: globular and with a blunt margin in *Ommatoiulus xerophilus*, and subtriangular in *Ommatoiulus chambiensis*; the paracoxite is apically more rounded in *Ommatoiulus chambiensis*, while the solenomerite has a notch and a distal pointed process in the latter species. With regard to somatic traits, *Ommatoiulus chambiensis* and *Ommatoiulus xerophilus* display differences in colour patterns and body size. *Ommatoiulus xerophilus* has clear pale dorsal longitudinal stripes which are lacking in *Ommatoiulus chambiensis*. Analysis of the variation of the maximum vertical diameter in relation to the number of podous rings for the different developmental stadia demonstrated that *Ommatoiulus chambiensis* is generally larger (more podous rings, higher body diameter) than *Ommatoiulus xerophilus* (Fig. 40). Of special importance is the fact that the size difference is apparent within each developmental stadium. Thus, *Ommatoiulus chambiensis* males with 8RO have more podous rings and larger diameter than *Ommatoiulus xerophilus* males with 8RO. The same is true for specimens with 9RO although the studied material is much smaller.

**Figure 41.**
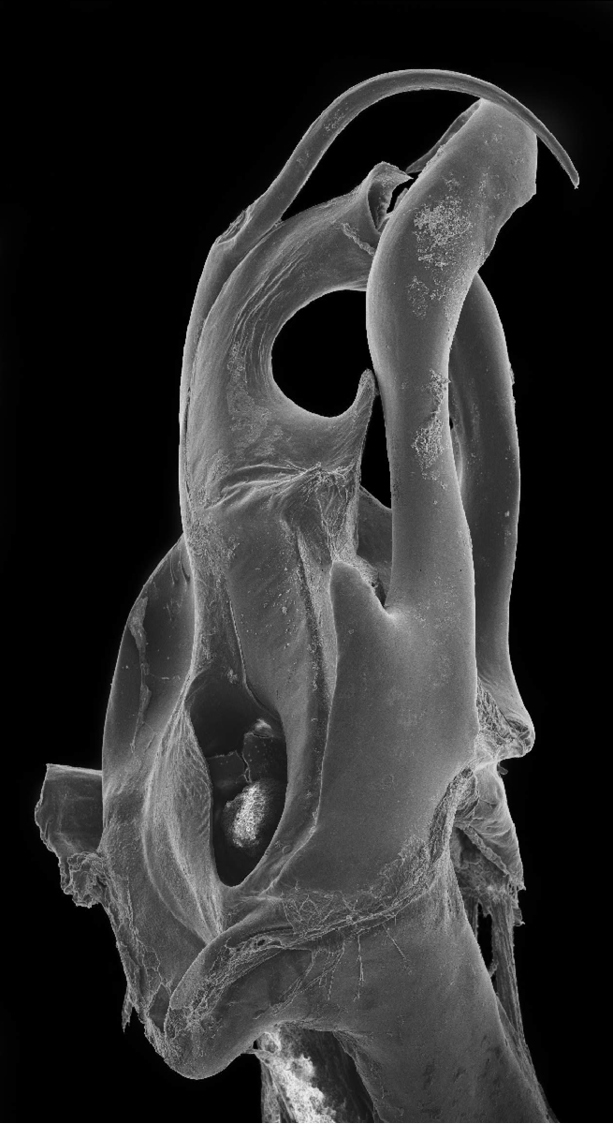
*Ommatoiulus punicus*,specimen from Jebel Rsas, NE Tunisia, right posterior gonopod. Interactive SEM image. [Morphbank # 831228–831245]

**Figure 42.**
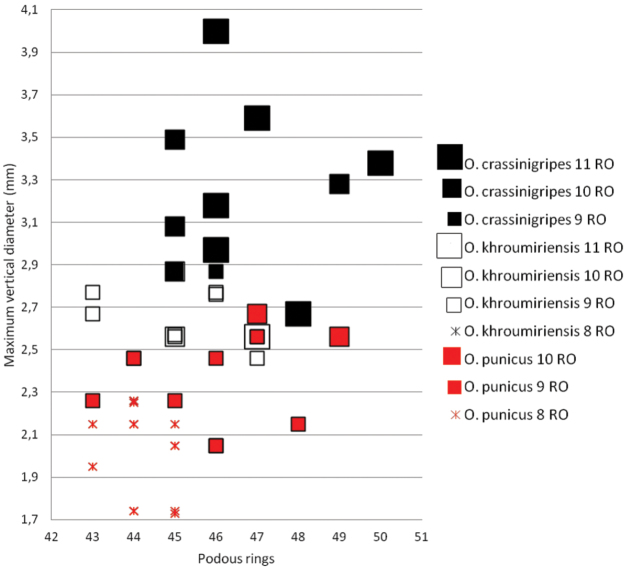
*Ommatoiulus crassinigripes* sp. n., *Ommatoiulus khroumiriensis* sp. n. and *Ommatoiulus punicus*. Scatter diagram illustrating size of adult males expressed as maximum vertical body diameter (mm) vs number of podous rings in different developmental stadia (8–11RO).

*Ommatoiulus crassinigripes* is the only schizophylline millipede collected at more than 1500 m elevation in Tunisia. However, it occurs also at lower elevation in central Tunisia (El Kef, Thala, Makthar, Kairouan) and is very unlikely to be an alticolous species like *Ommatoiulus gravieri* (Brolemann, 1924), which is known from 3000–3200 m altitude in Jebel Tachdirt of the High Atlas in Morrocco (Brolemann 1924, Akkari et al. 2009). *Ommatoiulus crassinigripes* is one of the largest *Ommatoiulus* species in Tunisia and, together with *Ommatoiulus khroumiriensis*, is the closest to *Ommatoiulus punicus* with regard to gonopod shape (Fig. 41). The three species, which were all treated under *Ommatoiulus punicus* by Akkari et al. (2009), have quite similar gonopod conformations, yet display a number of constant differences in both promerite and posterior gonopods (Table 1). Although resembling, differences in posterior gonopods can be clearly noticeable on the rotatable models (Figs 13, 26, 41), which, in addition to the external morphological features, indicate their separate taxonomic status. The external morphological differences concern colour pattern and size. Fig. 42 illustrates the variation of the maximum vertical diameter in relation to the number of podous rings. It clearly demonstrates that males of *Ommatoiulus crassinigripes* have more podous rings and are generally thicker compared to males of *Ommatoiulus punicus* and *Ommatoiulus khroumiriensis* of the same development stadium (with the same number of RO). The same applies when one compares males of stadia 9 and 10 RO of *Ommatoiulus punicus* and *Ommatoiulus khroumiriensis* where the latter is clearly thicker. The three species are allopatric in Tunisia and exhibit different habitat preferences. *Ommatoiulus khroumiriensis* is confined to the cork-oak forests of the Khroumirie in the northwest (mainly the Aïn Draham area), *Ommatoiulus crassinigripes* occurs in open, semi-arid habitats of the centre, in the plain of Kairouan and mountains of the Ridge, while *Ommatoiulus punicus* is mainly distributed around Tunis City, Cap Bon Peninsula and the eastern part of the Ridge *viz*. Zaghouan Mt. in the north (see Akkari et al. 2009). *Ommatoiulus punicus* was recorded from Aïn Draham, Tabarka and Nefza, as well as from Kairouan Plain by Akkari et al. (2009). These authors pointed out the variation observed in the material: “*Ommatoiulus punicus* as currently delimited is quite a variable taxon, and a detailed analysis may well necessitate splitting it into several (sub)species.” Our renewed study has corroborated this assumption. Attems (1903, 1926) must have been looking at *Ommatoiulus crassinigripes* and *Ommatoiulus khroumiriensis* while recording and illustrating *Ommatoiulus punicus*. In fact the gonopod drawings he provided left no doubt that what herecorded from Aïn Draham (Attems 1926, figs 240–241) is *Ommatoiulus khroumiriensis*. On the other hand, the record of *Ommatoiulus punicus* from unspecified locality in Tunisia Attems (1903: 144, figs 77–81) is here referred to as *Ommatoiulus crassinigripes*.

**Table 1.** Comparison of main gonopod and peripheral structures of *Ommatoiulus punicus*, *Ommatoiulus crassinigripes* and *Ommatoiulus khroumiriensis*.<br/>

	***Ommatoiulus punicus*<br/> (Figs 41, 51–53)**	***Ommatoiulus crassinigripes*<br/> (Figs 7–13)**	***Ommatoiulus khroumiriensis*<br/> (Figs 20–26)**
**Gonopod structures**	**Similarities**		
Promerite	abruptly narrowing in the distal half-third		
	deep lateral incision		
	mesal ridge protruding in a distal process		
Mesomerite	broad, distally narrowing and hook-shaped		
Solenomerite	broad, narrowing at mid-length		
	subapical triangular process		
	apical folded lamella		
	long and curved seminal groove		
Paracoxite	lamellar, folded, marginally serrated		
	**Differences**		
Promerite	lateral margin well rounded	lateral margin angular	lateral margin reduced and pointed
	distal process broad and triangular	distal process laterally rounded and bulgy	distal process slender, slightly expanded laterally
Mesomerite	distally slightly broaden and bearing serrations on the lateral margin	distally broaden latero-mesad and bearing strong serrations on both margins	distally strongly constricted and narrowed
Solenomerite			
Paracoxite	broad, apical margin rounded	broader, apical margin truncate	slender, apically tapering
**Non gonopod structures (males)**			
Body vertical diameter (mm)	1.7–2.7	2.7–3.6	2–2.8
Podous rings	43–48	45–53	43–48
Apodous rings	1–2	1–2	1–2
Colour	ash-grey alternating with light brown, legs purple	pale grey alternating with golden brown, legs black	dark grey alternating with yellowish brown, legs yellowish brown

Intercalary males are here recorded for the species *Ommatoiulus khroumiriensis* and *Ommatoiulus crassinigripes*. This is of little surprise considering that postembryonic development involving periodomorphosis (regression of secondary sexual characters followed by a return to a morphologically copulatory stadium following an additional moult) is considered as particularly prevalent for schizophylline species (Enghoff et al. 1993). Characteristic morphology of intercalary stadia was directly observed on reared specimens of three schizophyllines: *Ommatoiulus moreleti* (Lucas, 1860), *Ommatoiulus sabulosus* (Linnaeus, 1758) and *Tachypodoiulus niger* (Leach, 1814) while field-collected samples provided periodomorphic specimens belonging to no less than 8 species of genus *Ommatoiulus*, including *Ommatoiulus punicus* (see Akkari and Enghoff 2011 for species list). The same authors discussed the particular case of the species *Ommatoiulus sempervirilis* for which a large hand-collected sample revealed the complete absence of intercalary stadia and presence instead of four successive stadia of adult males implying a direct copulatory-copulatory succession (Akkari and Enghoff 2011).

Colour pattern and somatic characters cannot be used reliably to distinguish the Tunisian *Ommatoiulus* species, although we have provided information about these features above in order to point out fine differences between morphologically close or syntopic taxa. In the identification key (below) we prefer to include only gonopod characters.

### Identification key to Tunisian *Ommatoiulus* species

The key is based on characters of the male gonopods.

**Table:** 

1	Promerite with parallel margins, apically regularly rounded, without lateral incision (Fig. 43); mesomerite distally expanded into a subrectangular apical plate, concave and mesally incised (Fig. 44)	*Ommatoiulus sempervirilis*
–	Promerite with at least one lateral or apical incision (Figs 2, 7, 14, 20, 32, 45, 47, 49, 51); mesomerite different	2
2	Mesomerite (Ms) apically expanded and bifurcated (Figs 16, 46)	3
–	Mesomerite (Ms) apically narrowed and simple (Figs 3, 9, 24, 33, 48, 50, 53)	4
3	Mesomerite (Ms) hammer-shaped (Fig. 46), bifurcated into long asymmetrical processes; promerite (Fig. 45) broad, with a complex distal process, apically bearing two blunt bumps, a pointed process and a lateral serrated lamella	*Ommatoiulus malleatus*
–	Mesomerite (Ms) horn-shaped (Fig. 16), bifurcated into short and curved processes; promerite (Fig. 14) apically with three lobes separated by deep incisions	*Ommatoiulus kefi* sp. n.
4	Promerite gradually narrowing distally, lateral incision shallow to moderately deep; mesal ridge sometimes protruding in a serrated subapical process (se) (Figs 2, 32, 47); paracoxite (Px) without conspicuous marginal serrations (Figs 5, 34, 48, 50)	5
–	Promerite strongly narrowing distally, lateral incision deep; mesal ridge protruding in a blunt subapical process (Figs 7, 20, 51); paracoxite (Px) broad, lamellar, folded, with strong marginal serrations (Figs 8, 22, 52)	10
5	Mesomerite (Ms) slender, sinuous and strongly curved distally; solenomerite (S) with a long distal process (ds) lodging the seminal groove (Fig. 48)	*Ommatoiulus seurati*
–	Mesomerite (Ms) broad, distally narrowing; solenomerite (S) with a shorter distal process lodging the seminal groove (Figs 3, 33, 50)	6
6	Mesomerite (Ms) with irregular margins, apically strongly narrowed and hook-shaped; solenomerite (S) with a short distal process (ds) (Figs 37, 50)	7
–	Mesomerite (Ms) with a regular mesal margin, distally gradually narrowed into a bent folded process; solenomerite (S) with a longer distal process (ds) (Figs 3, 33)	8
7	Mesomerite with a subapical serrated lateral process, a truncated mesal margin, apically strongly narrowed into a small hook-shaped process (Fig. 50); promerite with a shallow lateral incision and a rounded distal process (Fig. 49)	*Ommatoiulus fuscounilineatus*
–	Mesomerite with a subapical lateral process (Figs 37, 39), apically gradually narrowed in a long curved process (Fig. 39); promerite with a deeper lateral incision and a broad distal process with apical and lateral lobes (Fig. 36)	*Ommatoiulus zaghouani* sp. n.
8	Promerite straight, bearing a pointed distal process (Fig. 27); paracoxite stout and shorter (Fig. 30)	*Ommatoiulus xenos* sp. n.
–	Promerite strongly bent anteriad, bearing a subapical serrated process (Figs 1, 31); paracoxite broad and elongate (Figs 5, 34)	9
9	Solenomerite (S) with a rounded notch (n), a pointed process (pr) and apically a wrinkled protruding lamella (Fig. 5); promerite with a subtriangular apex (Fig. 2)	*Ommatoiulus chambiensis* sp. n.
–	Solenomerite (S) without such a process; wrinkled lamella not protruding (Fig. 33); promerite with a globular apex (Fig. 32)	*Ommatoiulus xerophilus* sp. n.
10	Lateral incision of promerite extending basad, distal process broad and triangular (Fig. 20); mesomerite (Ms) distally strongly narrowed, without subapical serrations on lateral margin, paracoxite (Px) slender (Fig. 22)	*Ommatoiulus khroumiriensis* sp. n.
–	Lateral incision of promerite not extending basad, distal process slenderer and more rounded (Figs 7, 51); mesomerite (Ms) with lateral subapical serrations, paracoxite (Px) broader (Figs 12, 53)	11
11	Apical process of promerite (ap) bulgy, with a lobed process (ap1) on tip (Fig. 7); mesomerite (Ms) with strong subapical mesal serrations (Fig. 8); paracoxite (Px) broadened distally with truncated apex (Fig. 12)	*Ommatoiulus crassinigripes*
–	Apical process of promerite slender, without a lobed process; mesomerite (Ms) without mesal serrations (Fig. 52); paracoxite (Px) distally narrowed, with rounded apex (Fig. 53)	*Ommatoiulus punicus*

**Figures 43–48.**
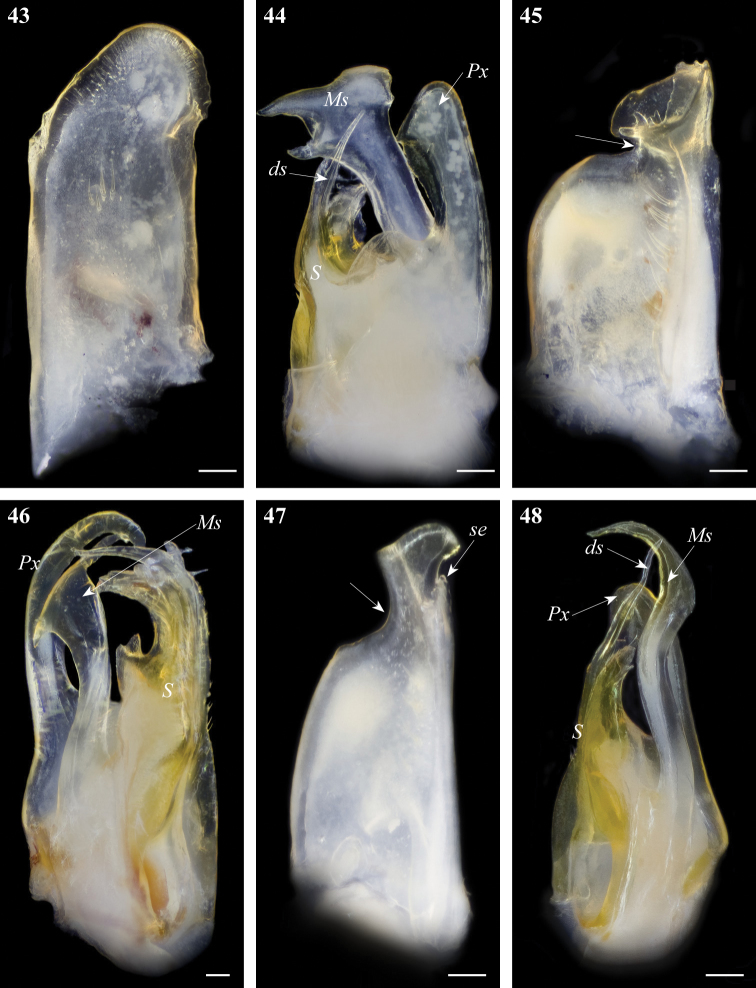
Gonopod structures of *Ommatoiulussempervirilis*, *Ommatoiulus malleatus*, *Ommatoiulus seurati* **43** *Ommatoiulus sempervirilis* left promerite, posterior view **44** *Ommatoiulus sempervirilis*, left posterior gonopod, meso-posterior view **45** *Ommatoiulus malleatus*, right promerite, anterior view **46** *Ommatoiulus malleatus*, left posterior, mesal view **47** *Ommatoiulus seurati* right promerite, posterior view **48** *Ommatoiulus seurati*, right posterior gonopod, antero-mesal view. Abbreviations: **ds** distal process of the solenomerite, **Ms** mesomerite, **Px** paracoxite, **S** solenomerite, **se** serrations. Arrow pointing to the lateral incision of the promerite. Scale bar 0.1 mm.

**Figures 49–53.**
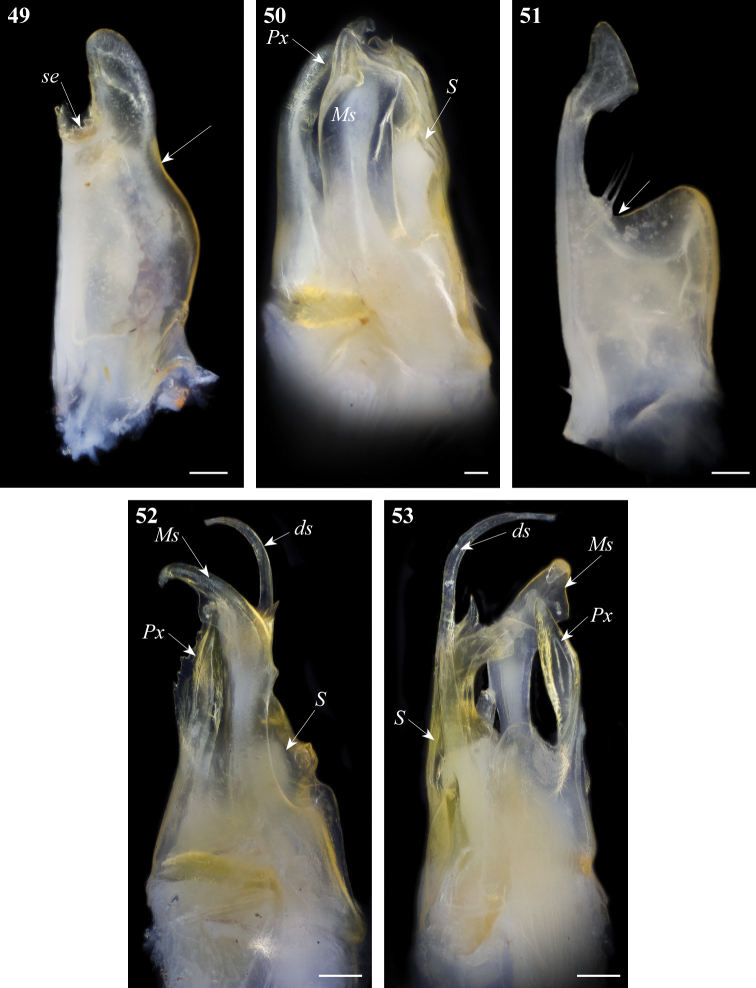
Gonopod structures of *Ommatoiulus fuscounilineatus*, *Ommatoiulus punicus*. **49** *Ommatoiulus fuscounilineatus*, left promerite, posterior view **50** *Ommatoiulus fuscounilineatus*, left posterior gonopod, anterior view **51** *Ommatoiulus punicus*, left promerite, posterior view **52** *Ommatoiulus punicus*,left posterior gonopod, lateral view **53** *Ommatoiulus punicus*,left posterior gonopod, posterior view. Abbreviations: **ds** distal process of the solenomerite, **Ms** mesomerite, **Px** paracoxite, **S** solenomerite, **se** serrations. Arrow pointing to the lateral incision of the promerite. Scale bar 0.1 mm.

### Interactive key to Tunisian *Ommatoiulus* species

We provide an interactive key in Flash (SWF) format to the *Ommatoiulus* species known from Tunisia. The key is dichotomous and based on gonopod characters. These are illustrated with line drawings, light microscopy photographs and SEMs, and for some of the species with rotatable SEM animations. A species list and species pages are included in the key to provide additional information on species diagnostic characters, distribution and habitats. An introductory section for the first-time user provides background information of importance for applying the key.

Adobe Flash Player (version 11.2 or higher) or a browser (e.g. Internet Explorer, Firefox and Chrome) with Flash Player plug-in is required to run the key.

## Supplementary Material

XML Treatment for Ommatoiulus

XML Treatment for Ommatoiulus chambiensis

XML Treatment for Ommatoiulus crassinigripes

XML Treatment for Ommatoiulus kefi

XML Treatment for Ommatoiulus khroumiriensis

XML Treatment for Ommatoiulus xenos

XML Treatment for Ommatoiulus xerophilus

XML Treatment for Ommatoiulus zaghouani

## Appendix 1

Source image library of the interactive SEM image of *Ommatoiulus chambiensis* (doi: 10.3897/zookeys.328.5763.app1) File format: WinZip Image Archive (jpg).

## Appendix 2

Source image library of the interactive SEM image of *Ommatoiulus crassinigripes* (doi: 10.3897/zookeys.328.5763.app2) File format: WinZip Image Archive (jpg).

## Appendix 3

Source image library of the interactive SEM image of *Ommatoiulus kefi* (doi: 10.3897/zookeys.328.5763.app3) File format: WinZip Image Archive (jpg).

## Appendix 4

Source image library of the interactive SEM image of *Ommatoiulus khroumiriensis* (doi: 10.3897/zookeys.328.5763.app4) File format: WinZip Image Archive (jpg).

## Appendix 5

Source image library of the interactive SEM image of *Ommatoiulus xerophilus* (doi: 10.3897/zookeys.328.5763.app5) File format: WinZip Image Archive (jpg).

## Appendix 6

Source image library of the interactive SEM image of O*mmatoiulus punicus* (doi: 10.3897/zookeys.328.5763.app6) File format: WinZip Image Archive (jpg).

## Appendix 7

Source file library of the interractive key to genus *Ommatoiulus* in Tunisia (doi: 10.3897/zookeys.328.5763.app7) File format: WinZip Archive (zip).
